# Real-time detection of 20 amino acids and discrimination of pathologically relevant peptides with functionalized nanopore

**DOI:** 10.1038/s41592-024-02208-7

**Published:** 2024-03-05

**Authors:** Ming Zhang, Chao Tang, Zichun Wang, Shanchuan Chen, Dan Zhang, Kaiju Li, Ke Sun, Changjian Zhao, Yu Wang, Mengying Xu, Lunzhi Dai, Guangwen Lu, Hubing Shi, Haiyan Ren, Lu Chen, Jia Geng

**Affiliations:** 1 Department of Laboratory Medicine, State Key Laboratory of Biotherapy and Cancer Center, Clinical Laboratory Medicine Research Center, West China Hospital, Sichuan University, Chengdu, China; 2 Biosafety Laboratory of West China Hospital, West China Hospital, Sichuan University, Chengdu, China; 3 Key Laboratory of Birth Defects and Related Diseases of Women and Children of MOE, Department of Laboratory Medicine, State Key Laboratory of Biotherapy, West China Second University Hospital, Sichuan University, Chengdu, China; 4 National Clinical Research Center for Geriatrics and Department of General Practice, State Key Laboratory of Biotherapy, West China Hospital, Sichuan University, Chengdu, China; 5 West China Hospital Emergency Department (WCHED), State Key Laboratory of Biotherapy, West China Hospital, Sichuan University, Chengdu, China; 6 Laboratory of Tumor Targeted and Immune Therapy, Clinical Research Center for Breast, State Key Laboratory of Biotherapy, West China Hospital, Sichuan University and Collaborative Innovation Center, Chengdu, China; 7 Division of Respiratory and Critical Care Medicine, State Key Laboratory of Biotherapy, West China Hospital of Sichuan University, Chengdu, China; 8 Tianfu Jincheng Laboratory, City of Future Medicine, Chengdu, China

**Keywords:** Nanopores, Protein sequencing, Single-molecule biophysics

## Abstract

Precise identification and quantification of amino acids is crucial for many biological applications. Here we report a copper(II)-functionalized *Mycobacterium smegmatis* porin A (MspA) nanopore with the N91H substitution, which enables direct identification of all 20 proteinogenic amino acids when combined with a machine-learning algorithm. The validation accuracy reaches 99.1%, with 30.9% signal recovery. The feasibility of ultrasensitive quantification of amino acids was also demonstrated at the nanomolar range. Furthermore, the capability of this system for real-time analyses of two representative post-translational modifications (PTMs), one unnatural amino acid and ten synthetic peptides using exopeptidases, including clinically relevant peptides associated with Alzheimer’s disease and cancer neoantigens, was demonstrated. Notably, our strategy successfully distinguishes peptides with only one amino acid difference from the hydrolysate and provides the possibility to infer the peptide sequence.

## Abstract (web summary)

A copper(II)-functionalized *Mycobacterium* *smegmatis* porin A nanopore enables direct identification of all 20 proteinogenic amino acids, one unnatural amino acid and two post-translational modifications, and shows potential for peptide discrimination and sequencing.

## Main

Amino acids are the building blocks of proteins. They are raw materials for biosynthesis and have fundamental roles in various physiological and pathophysiological processes, such as epigenetic regulation and tumor metabolism^1–4^. Therefore, it is crucial to detect and identify amino acids with a high spatiotemporal resolution, especially in the field of single-molecule protein sequencing^5–8^. Owing to alternative RNA splicing and PTMs, the resulting proteoforms are highly complicated and contain deeper-level information that cannot be accessed directly from the transcriptome^9^. In addition, there is no existing method similar to DNA amplification for amplifying proteins. Consequently, it is difficult to use mass-spectrometry-based methods to identify low-abundance proteins from the proteome^10,11^. To address these problems, single-molecule sequencing methods that can distinguish the 20 proteinogenic amino acids are needed.

Fluorophore-based techniques allow specific amino acids, such as cysteine and lysine, to be selectively modified by fluorescent molecules. Then, by sequentially degrading the peptide using Edman chemistry, or direct imaging using single-molecule fluorescence resonance energy transfer (FRET), the relative position of labeled amino acids can be deduced from the fluorescent signals^12–14^. Additionally, fluorophore-labeled amino-terminal recognizers of amino acids have been engineered to bind specific amino acids reversibly^15,16^. The repetitive signals of the same amino acid can greatly improve the accuracy of single-molecule peptide identification^17^. Although these methods have high throughput and reliability, it is difficult for chemists to label the 20 amino acids. For label-free methods, techniques such as tunneling current measurement^18,19^ and molecular junctions^20^ enable rapid, precise detection of up to 12 amino acids, which is still not sufficient for protein sequencing.

Given that the nanopore technique has demonstrated its superiority in single-molecule DNA sequencing, it is considered to be an ideal candidate for amino acid detection and protein sequencing^5,21,22^. Studies have shown that peptides with different properties, such as molecular weight^23,24^, length^25,26^, PTMs^27,28^ and single-amino acid substitutions^29^, can be detected directly and distinguished using nanopores. For further analysis of the peptide sequence, peptide translocation must be precisely controlled to generate sequence-dependent signals. The protein unfoldase ClpX has been used to unfold proteins and drive them through a nanopore, successfully discerning different protein segments^30^. Electro-osmotic flow can be engineered to facilitate unidirectional translocation of peptides with a heterogeneous charge distribution^31,32^. Moreover, the ratcheting motion of DNA–peptide conjugation through the nanopore has been achieved using DNA helicase or polymerase, generating clear sequence-dependent signals^33–35^. However, there are 20 types of amino acid, so deconvoluting the signals produced by 5–6 amino acids is more complex than analyzing the signals from the four types of nucleotide, because there are many more possible combinations of amino acids. Consequently, the analysis of individual amino acids can provide valuable information and could be an alternative to peptide sequencing. Taking advantage of the pore structure, the aerolysin nanopore can differentiate 13 out of 20 amino acids when coupled with a polyarginine carrier^36^. Furthermore, copper-ion-modified α-hemolysin and the solid-state MoS_2_ nanopore have been developed to detect underivatized amino acids^37,38^. Most recently, the MspA-NTA nanopore with a Ni^2+^ modification has been able to distinguish the 20 proteinogenic amino acids and their PTMs with high resolution^39^. Meanwhile, an exopeptidase protein-sequencing method in which amino acids were coupled to the peptide probe FGGCD_8_ through a chemical linker was developed using an α-hemolysin nanopore^40^. It enables an integrated approach to peptide sequencing. However, real-time detection of cleaved amino acids during peptide hydrolysis has not yet been achieved, hampering the development of single-molecule peptide sequencing.

Here, we report the direct detection of 20 proteinogenic amino acids using a copper(II)-functionalized MspA nanopore, with the limit of detection at the nanomolar range. We introduced histidine substitutions in the constriction region of the pore lumen to construct the binding sites for copper ions. With the copper ion binding to histidine residues, the reversible coordination between amino acid and copper–nanopore complex could generate well-defined current signals, enabling the detection of all 20 proteinogenic amino acids, 2 amino acids with PTMs (*O*-phosphoryl-l-serine (P-S) and Nε-acetyl-l-lysine (Ac-K)) and 1 unnatural amino acid (*S*-carboxymethyl-l-cysteine (CMC)). Furthermore, by analyzing the composition of peptide hydrolysate using exopeptidase, we identified ten different peptides. Our method enables the real-time detection of the cleaved amino acids during peptide hydrolysis and offers the possibility of inferring peptide sequences.

## Results

### Sensing of 20 proteinogenic amino acids

**Fig. 1:**
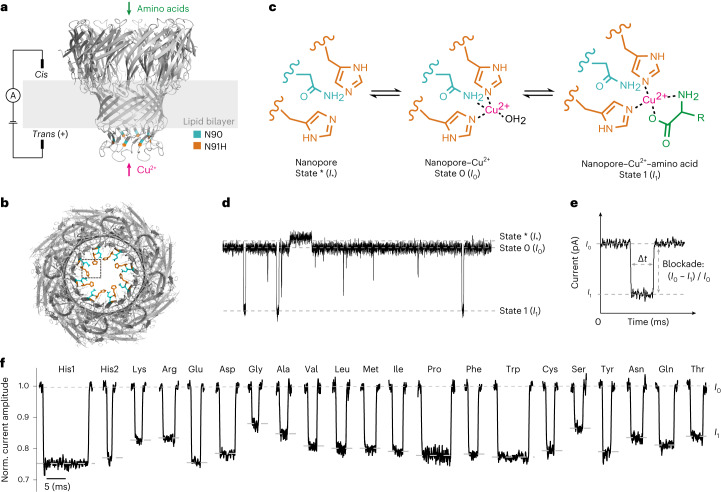
Experimental setup and principle of amino acid detection. **a**, Schematic of the experimental setup. Amino acids and copper ions were added to the *cis* and *trans* chambers, respectively. A voltage of +50 mV was applied during measurement. The N91H substitutions in eight subunits are highlighted in orange. **b**, Bottom-view structure of the MspA-N91H nanopore (predicted using SWISS-MODEL). The dotted box shows a binding site for the copper ion. **c**, Proposed sensing mechanism. Two adjacent histidine residues (position 91) and one asparagine residue (position 90) coordinate a copper ion. Then, the α-amine and α-carboxyl groups of the amino acid coordinate the copper–histidine complex. **d**, A representative current trace showing the corresponding current change for three binding states. **e**, Illustration of current blockade induced by the binding of amino acid. **f**, Representative signals of current blockade events of 20 amino acids. The events of histidine exhibited two populations, His1 and His2. Source data

The conical pore geometry of the MspA nanopore makes it an ideal choice for examining small molecules^41,42^. However, a previous study has demonstrated that copper modification of the α-hemolysin nanopore enables the detection of four proteinogenic amino acids^37^. Therefore, a copper-modified MspA nanopore could potentially exhibit higher sensitivity for amino acids. To this end, we designed the MspA-N91H nanopore and tested whether it can coordinate copper(II) and amino acids in a typical single-channel recording setup (Fig. 1a). For each subunit of the octameric nanopore, the asparagine at position 91 is substituted by histidine. This substitution is located at the constriction region of the nanopore. Together with the asparagine residue at position 90, a copper-binding structure can be created (Fig. 1b). The structure is similar to the histidine brace motif^43^. We hypothesized that one asparagine residue at position 90 and two adjacent histidine residues at position 91 from two subunits could reversibly coordinate one copper ion and one amino acid molecule (Fig. 1c). After adding the amino acid and Cu^2+^ into the *cis* chamber (electrically grounded) and *trans* chamber, respectively, of a pair of electrolytic chambers, the binding of different molecules can be observed from the current trace (Fig. 1d). In the current trace, the current of a single nanopore in the open state is denoted as I_*_ (state *). The states 0 and 1 represent the stable state after the binding of copper ions and the state after the binding of one amino acid molecule, respectively.

To validate our hypothesis, we performed three control experiments. First, we demonstrated that wild-type MspA cannot coordinate copper ions and an amino acid (Supplementary Fig. 1). Second, without copper ions, amino acids cannot be detected using MspA-N91H (Supplementary Fig. 2). Third, neither acetylated leucine nor amidated leucine generated distinguishable signals with copper-modified MspA-N91H (Supplementary Fig. 3), indicating that the copper–histidine complex of the nanopore coordinates amino acids’ α-carboxyl group and α-amine groups. The reversible binding of multiple copper ions was observed, owing to the four binding sites at the constriction region^44^. Such stochastic binding events interferes with the precise assay of subsequent amino acid binding. To keep the current baseline at the stable state (state 0 in Fig. 1c,d), excess copper ions (with a final concentration of 200 μM) were added into the *trans* chamber to saturate the binding sites during most of the measuring time (approximately 87.8 ± 3.1%) (Supplementary Table 1).

**Fig. 2:**
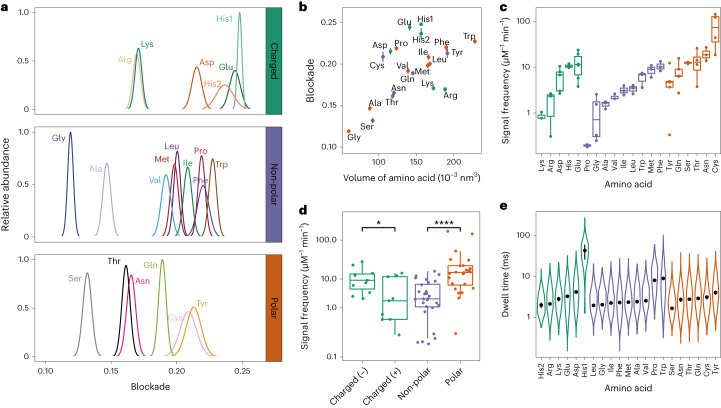
Characteristics of signals of the 20 proteinogenic amino acids. **a**, The distribution of relative abundance of amino acid signal blockades. *n* = 4,278 (E), 4,211 (D), 650 (K), 193 (His1), 306 (His2), 7,166 (F), 3,934 (W), 2,768 (Y), 3,025 (I), 8,004 (M), 3,059 (R), 8,131 (T), 8,101 (S), 3,750 (L), 857 (A), 1,149 (G), 361 (P), 7,873 (Q), 9,634 (N), 2,119 (V), 616 (C). **b**, Mean blockade versus volume of amino acids. For each amino acid, the mean blockade and its s.d. were calculated from the Gaussian fitting result of histogram of blockade. The numbers of analyzed events are identical to those in **a**. Amino acids with a charged side chain, a non-polar side chain and a polar side chain are colored green, purple and orange, respectively. **c**, Signal frequency of amino acids. Each dot represents data from an independent experiment. **d**, Signal frequency of four categories of amino acids. The signal frequency of amino acids with a polar side chain is significantly higher than that of amino acids with a non-polar side chain. Statistical analysis was done using the Wilcoxon rank-sum test (*P* = 0.04 and 1.73 × 10^–7^, two-sided). Midline, median; box limits, 25th (Q1) and 75th (Q3) percentiles; whiskers, Q3 + 1.5 × IQR and Q1 – 1.5 × IQR; IQR, interquartile range. The asterisks indicate the statistical significance (* and **** represent *P* ≤ 0.05 and *P* ≤ 0.0001, respectively). **e**, Mean dwell time of all identified signals for each amino acid. The points and whiskers represent the half-life and standard error (s.e.) calculated from the fitting of the exponential decay function. Number of independent experiments, *n* ≥ 3. The data are presented as mean ± s.d. for blockade and signal frequency, mean ± s.e. for dwell time. Source data

The binding event of one amino acid molecule generated the state 1 signal (Fig. 1e,f). The blockade ((*I*_0_ − *I*_1_) / *I*_0_) and dwell time (∆*t*) were calculated to characterize the signal; *I*_0_ was considered the current baseline (Supplementary Table 2). For each amino acid (except histidine), signal blockade exhibited unimodal distribution (Fig. 2a). However, overlap was observed between the blockades from several amino acids (Lys and Arg; Met and Leu; Pro and Phe; Thr and Asn; Cys and Tyr). To better distinguish the signals, a machine-learning-based classifier was developed (the results are discussed in the next section). A positive correlation between the mean blockade and amino acid volume was observed (Fig. 2b). Moreover, when cysteine, proline and amino acids with a charged side group are excluded, the Pearson correlation coefficient between the mean blockade and volume reaches up to 0.97 (Supplementary Fig. 4). This indicates that, for most amino acids, the current blockade obeys the classical volume exclusion model^45^. For amino acids with charged side groups, the volume exclusion model is no longer applicable, which has been reported previously^46^. The signals of histidine showed two different populations, which was also observed in a previous study with a Ni^2+^-modified MspA nanopore (Supplementary Figs. 5–7)^39^. It is also worth mentioning that the binding of copper ions to nanopore was extremely unstable when cysteine (Cys) was added (Supplementary Fig. 8). We hypothesize that the strong interaction between copper ions and the sulfhydryl group of cysteine interfered with the binding of copper ions to the nanopore. Therefore, we tested CMC with a modified sulfhydryl group. The addition of CMC did not cause abnormal current fluctuation (Supplementary Fig. 9).

There are remarkable differences among the amino acids in the frequency at which each of them is captured (Fig. 2c). This can be partly attributed to the different electrophoretic and electro-osmotic forces applied to them. Proline has the lowest signal frequency. This is because the secondary amino group of proline could be less advantageous for its binding to the copper–histidine complex. The mean signal frequency of amino acids with a polar side chain is significantly higher than is that of non-polar amino acids (Fig. 2d). The mean dwell time of amino acids is within the range of 1 to 10 ms, except for His1, whose mean dwell time is 42.7 ± 17.1 ms (Fig. 2e and Supplementary Table 2).

### Identification of amino acids by machine learning

**Fig. 3:**
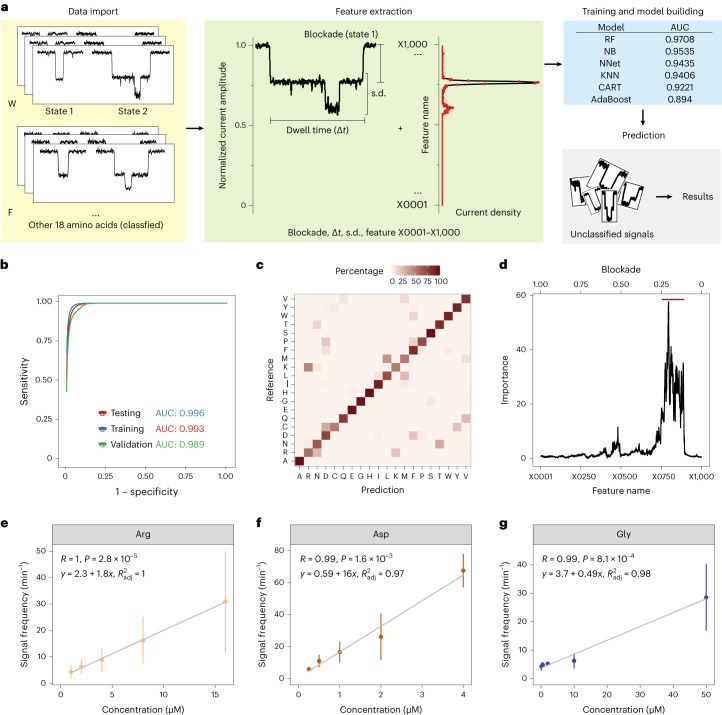
Amino acid identification assisted by a machine-learning algorithm. **a**, Illustration of the training process. First, signals corresponding to classified state 1 (one amino acid bound) and state 2 (two of the same amino acid bound) for each type of amino acid were imported and normalized. Then, the state 1 blockade, dwell time and s.d. were extracted. Additionally, 1,000 data points, named feature X0001–X1000, were extracted from the current density of each signal (from 0 to 1 with an interval of 0.001). Model performance was tested, including RF, NB, NNet, KNN, bagged CART and AdaBoost. RF outperformed the other models, achieving an AUC of 0.990. A tenfold cross-validation was used to prevent overfitting. **b**, The receiver operating characteristic curve (ROC) of the RF model for the training, testing and independent validation data sets of state 1 signals for all 20 amino acids. **c**, Confusion matrix of amino acid classification generated by the RF model using feature matrix. **d**, Feature importance generated from training of RF for state 1 signals of all 20 amino acids. The upper *x* axis represents the corresponding blockade of each feature. Features within the range of state 1 blockade of all amino acids have a higher importance value (marked by the red line). **e**,**f**,**g**, Scatter plot of signal frequency versus concentration of amino acids (Arg (**e**), Asp (**f**) and Gly (**g**)). The data are presented as mean ± s.d. The *R* and *P* values were calculated on the basis of Pearson correlation. The formulas and adjusted *R*^2^ values were computed on the basis of linear regression. *n* ≥ 3 independent experiments. Source data

To use signals for the identification of amino acids, we developed a machine-learning-based classifier, comprising three main steps: data import; feature extraction; and model training and construction (Fig. 3a). First, we randomly selected 1,000 events from the 20 amino acid types to form the training data set (Supplementary Fig. 10a). Second, to extract the feature from the current trace, we normalized the signals by *I*_0_ and then divided them into 1,000 equally sized intervals (Fig. 3a). Then, four event features (that is, the mean blockade, dwell time, s.d. and normalized signal density over 1,000 intervals) were used as an input matrix to train the classifier. Third, the feature matrix was passed to six classifiers for evaluation, including random forest (RF), naïveByes (NB), neural network (NNet), *k*-nearest neighbor (KNN), bagged classification trees (CART) and adaptive boosting classification trees (AdaBoost), given 100 signals for each amino acid. The RF model, which has an area under the curve (AUC) of 0.990 in the training data set, performed the best (Fig. 3a). When given 1,000 signals of each amino acid, the AUCs of the RF model were further increased to 0.996, 0.993 and 0.989 in the training, testing and validation data, respectively (Fig. 3b and Supplementary Fig. 10a,b).

Next, to evaluate the trade-off between accuracy and efficiency, we used different threshold values of prediction probability to filter prediction results. We found that the RF classifier can achieve 95.2% accuracy when using 43.1% of signals, and 99.1% accuracy when using 30.9% of signals, in the unlabeled validation set (Supplementary Fig. 10c,d). The confusion matrix result indicated that most amino acids can be distinguished from others (Fig. 3c and Supplementary Table 3). These results suggest that the MspA-N91H can identify amino acids with high accuracy.

When extracting the features, we noticed some multilevel signals (Supplementary Fig. 8), which could be beneficial for identifying signals of a certain amino acid (Supplementary Fig. 11 and Supplementary Discussion 1). We thus used our RF model to assess the importance of all the features including these multilevel signals. In this model, we identified that the blockades of all state 1 signals from 20 amino acids have larger importance values than those of state 2 signals (Fig. 3d). Because these multilevel signals could have resulted from noise or unknown integration of multiple amino acids, especially when different types of amino acids were mixed, we used only the state 1 signals in our machine-learning model.

Finally, to assess whether different types of amino acid can be discriminated simultaneously in a mixture, we added ten amino acids successively and analyzed the signals. We found that each amino acid in a mixture of ten proteinogenic amino acids (Gly, Ser, Ala, Thr, Arg, Gln, Met, Ile, Trp, Glu) and CMC can be discriminated precisely (Supplementary Fig. 12).

### Quantification of amino acids with high sensitivity

Given that our machine-learning method provided the counts of amino acid signals from the current traces, we assessed the relationship between the signal frequency and concentration of amino acids. Representative amino acids with a non-charged side chain (Gly), positively charged side chain (Arg) or negatively charged side chain (Asp) were tested individually at different concentrations. Strong positive correlations were consistently observed in these three amino acids (Fig. 3e–g; Pearson correlation, *R* > 0.99, *P* < 0.0011). We further used linear regression to establish a predictive formula between signal frequency and concentration for each amino acid (*R*^2^ > 0.97, linear regression), suggesting that our method can potentially quantify the concentration of amino acids within the micromolar range.

To test the sensitivity of our method, we used the definition of the limit of detection (LOD) in a previous study^39^, that is, the minimum concentration that enables the detection of more than five amino acid signals within a continuous 10-min recording of current. The LODs of amino acids tested in this study are 100 nM, 250 nM and 1 μM for Gly, Asp and Arg, respectively (Fig. 3e–g and Supplementary Figs. 13–15). The LOD of glycine (<100 nM) achieved by our method is at least 500 times lower than that (50 μM) in a similar study^39^. This LOD is much closer to the analyte concentration in cells. In summary, our method offers the possibility of quantifying amino acids with high sensitivity.

### Discrimination of unnatural and PTM amino acids

**Fig. 4:**
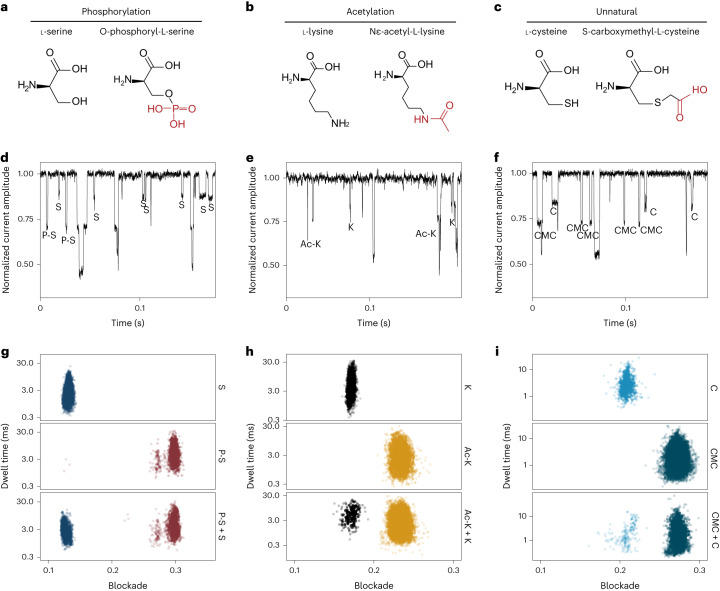
Identification of amino acids with PTMs and the unnatural amino acid. **a**–**c**, Chemical structure of the amino acids, from left to right: S, P-S (**a**), K, Ac-K (**b**), C and CMC (**c**). **d**–**f**, Representative current trace of events generated by simultaneous sensing of proteinogenic amino acids and their PTMs, or of the unnatural amino acid. Final concentrations of S and P-S were both 30 μM (**d**); of K and Ac-K were 200 μM and 100 μM, respectively (**e**); and of C and CMC were 3 μM and 20 μM, respectively (**f**). **g**–**i**, Blockade versus dwell time of events from the sensing of individual amino acids and the mixtures. Source data

PTMs, the breaking or generation of covalent bonds in the protein backbone or amino acid side chains, increase the complexity of the proteome in health and disease^47^. To evaluate the sensitivity of our method for PTM detection, we tested two amino acids with PTMs, P-S and Ac-K (Fig. 4a,b). Notably, we utilized the same nanopore for profiling both amino acids and their modifications, generating signals with distinct characteristics (Fig. 4d,e). In the corresponding scatter plots, each pair of results exhibited two distinct signal clusters, clearly differentiated from one another (Fig. 4g,h). The blockade profiles for S and P-S were 0.132 ± 0.0033 and 0.295 ± 0.0093 (mean ± s.d.); for K and Ac-K, they were 0.171 ± 0.0026 and 0.233 ± 0.0071, respectively. These findings underscore the method’s potential applicability to other amino acids with PTMs.

The incorporation of unnatural amino acids with novel side chains into proteins introduces a new dimension to the study of protein structure and function. Previous research has involved the investigation of four derivatives of five amino acids using α-hemolysin nanopores^48^. To the best of our knowledge, no study has used nanopores to achieve simultaneous sensing of unnatural amino acids and their corresponding natural amino acids. In our study, we conducted experiments involving cysteine and CMC (Fig. 4c), and found that the CMC signals exhibited blockades that were notably different from those of cysteine signals (Fig. 4f,i). This outcome suggests that the MspA-N91H–copper complex could potentially be used to analyze other unnatural amino acids or amino acids containing PTMs, with high resolution and sensitivity.

### Real-time detection of amino acids during peptide hydrolysis

**Fig. 5:**
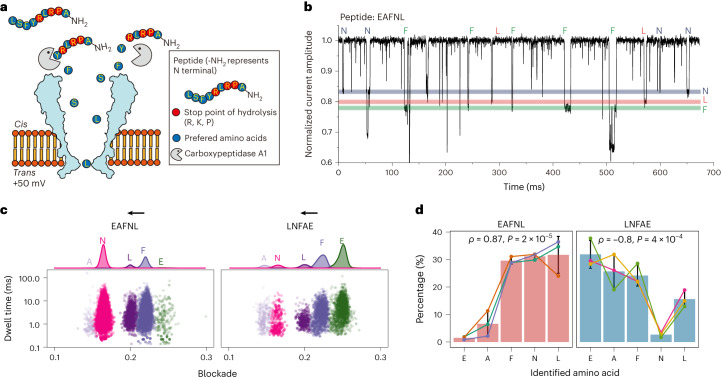
Real-time identification of amino acids during peptide hydrolysis. **a**, Schematic of the experiment. The peptide and carboxypeptidase A1 were added directly to the nanopore. Individual amino acids (except Arg, Lys and Pro) could be cleaved from peptides and detected. **b**, A representative current trace of amino acid signals during peptide hydrolysis. The target amino acids can be identified correctly from the normalized current amplitude. **c**, Scatter plots of two peptides (EAFNL and LNFAE) after hydrolysis with reversed peptide sequences. The black arrows represent the direction of hydrolysis. **d**, Mean abundance of identified amino acids from the two peptides. Results from each independent experiment are shown in different colors. *ρ* is the Spearman’s rank correlation coefficient, and *P* values were calculated from the Spearman’s rank correlation test. Hydrolysis and detection were performed in electrolyte buffer (1 M KCl, 10 mM MOPS, pH 7.5). Data are presented as mean ± s.d. *n* = 3 independent experiments. Source data

Because it is challenging to sequence a polypeptide directly, we assessed the feasibility of using our method to detect individual amino acids cleaved from peptides in real time. Peptide hydrolysis in the *cis* chamber was initiated by the addition of carboxypeptidase A1, without any additional sample processing. According to the substrate preference of carboxypeptidase A1, some amino acids from the carboxy terminus of the peptide can be cleaved, whereas amino acids such as R, K and P stop hydrolysis (Fig. 5a). To test our system, we first synthesized a peptide with sequence EAFNL. After the addition of carboxypeptidase A1, the signals of the expected amino acids (L, N and F) were observed (Fig. 5b), suggesting that the hydrolysis of the peptide in the chamber did occur. However, we found only a few signals for A and E, which are located in the N terminus. This result indicates that the hydrolysis, which started from the C terminus, might lead to more signals from amino acids closer to this terminus (in this case, L, N and F).

To investigate whether there is a trend toward higher abundance of amino acid signals closer to the C terminus, we performed experiments using two peptides with reversed sequences, EAFNL and LNFAE. Indeed, the distribution of identified amino acids during hydrolysis was remarkably different (Fig. 5c). For EAFNL, most of the signals belonged to the first three amino acids (L, N and F) from the C terminus. By contrast, most of the signals were identified as E and F in LNFAE, with only a few N and L signals.

To explore the possibility of inferring the sequence of peptides by taking advantage of this trend, we compared the abundance of each amino acid in these two peptides. Given that individual amino acids have different capture rates (Fig. 2c), the absolute signal count does not directly represent the abundance of cleaved amino acids. We thus normalized the count of signals by the mean capture rate for different amino acids and then standardized it to the percentage of amino acids in the hydrolysate. We observed a general increasing trend of the percentage of amino acid abundance toward the C terminus in both EAFNL and LNFAE (Fig. 5d; Spearman’s rank correlation coefficient = 0.87 and −0.8, respectively), except for the N in LNFAE. We reasoned that, owing to insufficient peptide hydrolysis, within the time of the detection, the amino acids closer to C terminus were more likely to be cleaved, resulting in a higher abundance. Therefore, although the composition of the two peptides is identical, we detected opposite patterns in terms of the abundance of amino acids during hydrolysis, with higher abundance of amino acids detected closer to the C terminus (Fig. 5d). Our results show that this strategy can be used to detect amino acids in real time from the C terminus, and this trend of signal abundance toward the C terminus might be used to infer the potential order of the peptide.

### Distinguishing amino acid replacements in peptides

To evaluate the viability of using nanopore sensing for the early detection or treatment of diseases through the identification of pathologically relevant peptides, we used our method to analyze synthetic peptides associated with Alzheimer’s disease (AD) and cancer neoantigens by investigating the different amino acid compositions of peptide hydrolysates.

**Fig. 6:**
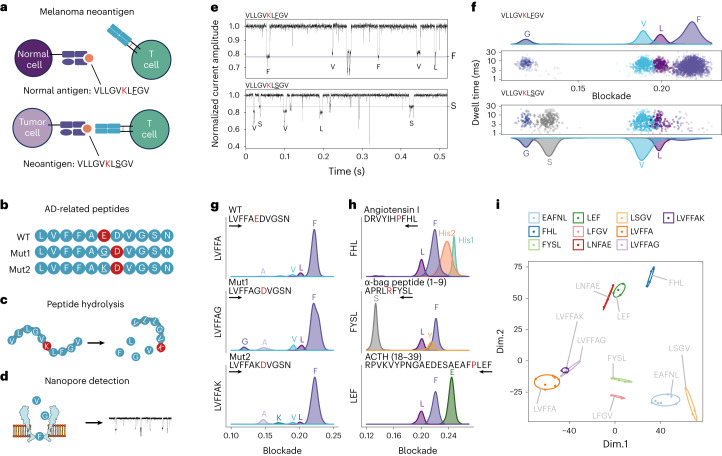
Distinguishing pathological peptides with amino acid substitutions. **a**,**b**, Schematic of the analysis and sequences of synthetic normal antigens and neoantigens of melanoma (**a**). Wild-type Aβ (linked to AD) and two mutants were synthesized (**b**). The amino acids shown in red represent the stop point of hydrolysis. **c**,**d**, The peptides were hydrolyzed separately using exopeptidases (**c**), and the released amino acids of each peptide were then detected separately using our nanopore sensor (**d**). **e**,**f**, Dwell time versus blockade of signals identified from peptide hydrolysate (**f**), and the corresponding current trace during detection (**e**). Top: normal antigen; bottom: neoantigen peptide. The hydrolysis started from the C terminus. **g**, Blockade of amino acid signals identified from peptide hydrolysate of three Aβ (17–27 aa) peptides. The black arrows represent direction of hydrolysis. **h**, Amino acid identification of the hydrolysate from angiotensin I, α-bag peptide (1–9 aa) and ACTH (18–39 aa). **i**, MDS of a Euclidean distance matrix (EDM) of all ten types of polypeptide. Source data

Neoantigens are cancer-specific peptides displayed on the cell surface and are caused by various tumor-specific alterations, such as mutation and dysregulated RNA splicing^49^. Neoantigens are emerging targets for personalized cancer immunotherapies and predictors for tumor survival prognosis and response to immune checkpoint blockade. Two primary strategies for identifying neoantigen epitopes are in silico predictions based on next-generation sequencing (NGS), and mass spectrometry (MS) for the analysis of major-histocompatibility-complex-loaded peptides^50^. Nanopore-based de novo sequencing of peptides could offer the possibility of direct neoantigen identification. To investigate its feasibility, we synthesized an HLA-A2-restricted neoantigen in COL18A1, that is, neoantigen peptide (VLLGVKLFGV) and its normal counterpart (VLLGVKLSGV), from a person with melanoma (Fig. 6a)^51^. After digestion of the peptides, the product was added to the nanopore for detection (Fig. 6c,d). The released amino acids can be differentiated by their distinct signal blockades. All the expected amino acids were identified, and the difference between the hydrolysates of the two peptides was observable (Fig. 6e,f).

AD is a neurodegenerative disease that affects millions of people. Point substitutions in the β-amyloid (Aβ) region of the amyloid precursor protein (APP) can lead to protein misfolding and aggregation, contributing to the onset of this disease. These substitutions account for 10–15% of early-onset familial AD cases and are thus considered the leading biomarkers for accurate and early diagnosis of AD^52,53^. Given that the identification of Aβ mutants is crucial for early diagnosis, we used our nanopore to analyze the clinically important wild-type Aβ peptide (17–27 amino acids (aa); ^17^LVFFAEDVGSN^27^) and two mutants (^17^LVFFAKDVGSN^27^ and ^17^LVFFAGDVGSN^27^), with a single amino acid difference (Fig. 6b). The peptides were hydrolyzed from the N terminus using aminopeptidase. Compared with the wild type, the hydrolysate of the two mutants (^17^LVFFAGDVGSN^27^ and ^17^LVFFAKDVGSN^27^) presented clear signals of G and K, respectively (Fig. 6g), suggesting that our strategy can correctly identify the amino acid replacement in these AD-associated peptides.

Next, to assess the generalizability of our method across a broader range of peptides, we purchased three commercially available products: angiotensin I, α-bag cell peptide (1–9 aa) and adrenocorticotropic hormone (ACTH; 18–39 aa), which are commonly used to investigate neurons, insulin secretion and the regulation of blood pressure, respectively. Using our method, the composition of the C terminus was identified correctly, and the hydrolysis was terminated at the expected stop points of carboxypeptidase A1 (Fig. 6h and Supplementary Fig. 16), suggesting that our method is robust in a variety of peptides.

Finally, we compared the similarities between all ten types of peptide using only their blockage distribution from each peptide, without the amino acid information (Supplementary Fig. 17). Then, we calculated the Euclidean distance according to the estimated density distribution of standardized current of all peptides, to evaluate peptide similarity (Supplementary Fig. 18). The classical multidimensional scaling (MDS) algorithm was used to get the best-fitting representation of the peptides using Euclidean distances. As shown in Fig. 6i, three AD-associated peptides were clustered together in the MDS plot (Fig. 6i, bottom right). Similarly, LEF and LNFAE, which have three common amino acids, were also clustered closely (Fig. 6i), whereas EAFNL and LNFAE, which have the same amino acids but in reverse sequence, were clustered distantly, suggesting that our profiling of peptides reflects the composition and sequence of the peptides and can be used for unsupervised clustering of peptide sequencing.

## Discussion

Using the interaction between the α-amine group and α-carboxyl group of amino acids and the copper–nanopore complex to generate current blockade, we developed a copper-ion-functionalized MspA nanopore. This nanopore sensor enables the identification of all 20 proteinogenic amino acids, 2 amino acids with PTMs (P-S and Ac-K) and one unnatural amino acid (CMC).

Recently, a Ni^2+^-modified nanopore^39^ has shown high accuracy, stability and robustness in the identification of amino acids. Meanwhile, an α-hemolysin nanopore that provides peptides’ identities and sequences with the assistance of a peptide probe has been developed^40^. Together with our study, these results suggest that there is a promising future in which nanopores can be used to achieve single-molecule protein sequencing. However, there are several key barriers. First, because the proteins cannot yet be amplified, the limit of detection is crucial for applications in sensing proteins with low abundance. Our method improves the sensitivity, and the LOD was within the nanomolar range (Fig. 3e–g). The LOD of Gly was below 100 nM in our study, compared with 50 μM when using the Ni^2+^-modified nanopore. Second, quantification along with the identification of amino acids using nanopores is still challenging. Promisingly, our RF-based machine-learning method not only can classify amino acids, but also has the potential to quantify the concentration of individual amino acids. Finally, through real-time detection of the released amino acids during peptide hydrolysis, we demonstrated that the hydrolysates of peptides with reversed sequences (EAFNL and LNFAE) exhibited opposite trends in the abundance of identified amino acid signals (Fig. 5c,d), indicating that our method provides clues to infer the likelihood of the sequence order of the targeted peptides. Compared with the strategy using α-hemolysin nanopores^40^, which requires multiple nanopores and several chemical steps for peptide identification, our real-time analysis is a faster, simpler solution.

Notably, we prove that, in principle, our strategy can distinguish the normal and mutant amino acids in AD peptides and neoantigens from melanomas. For future applications, the accuracy and efficiency of identifying amino acids in a mixture need to be improved. Nevertheless, this method offers more direct identification of peptides with amino acid resolution, compared with peptide fingerprinting^54^. We expect that a generic peptidase, such as carboxypeptidase Y, could be modified and conjugated to the top of the nanopore; the capture rate of cleaved amino acids could be further improved by engineering the electro-osmotic flow across the nanopore. Together with previous studies^36,39,40^, our work suggests that nanopore technology has the potential to provide sufficient resolution to identify and distinguish amino acids in real time, paving the way to protein sequencing, the comprehensive understanding of proteome and direct monitoring of disease status in the realm of proteins.

## Methods

### Protein nanopore preparation

MspA-N91H was expressed and purified as described previously^44^. In brief, the gene encoding M2MspA gene with a substitution at histine 91 was cloned into pET28b vector. Then, the plasmid was transformed by heat shock into *Escherichia coli* BL21 (DE3) competent cells. The cells were cultured in LB medium containing kanamycin (50 μg ml^–1^) to an optical density at 600 nm of 0.8, and then 1 mM isopropyl β-d-1-thiogalactopyranoside (IPTG) was added. Afterward, cells were incubated at 15 °C for 12 h with shaking at 220 rpm. Then, the cells were collected by centrifugation at 5,180*g*, 4 °C for 15 min and re-resuspended. Cell disruption was performed by sonication using an ultrasonic cell disruption device. The supernatant was retained, and the target protein was further purified using an anion exchange column (Q-Sepharose) and size-exclusion column (Superdex 200 16/90).

### Detection of proteinogenic, unnatural and PTM amino acids

Electrophysiology experiments were performed using a classical vertical lipid bilayer setup with a lipid membrane that separates a pair of chambers filled with electrolytic fluid (Warner Instruments). A pair of Ag and AgCl electrodes was placed in the *trans* and *cis* (grounded) side of the chamber, which was filled with 1 ml of electrolyte solution (1 M KCl, 10 mM MOPS, pH 7.5). Then, the planar lipid bilayer membrane was formed on the 150 μm-diameter aperture by painting a thin film of 1,2-diphytanoyl-*sn*-glycero-3-phosphocholine (DPhPC) (Avanti Polar Lipids). A voltage of +300 mV was applied to induce nanopore insertion after the MspA protein was added (final concentration of 60–90 ng ml^–1^) into the *cis* chamber. After a single nanopore insertion, CuCl_2_ solution was added into the *trans* chamber to a final concentration of 200 μM (20 μM in peptide hydrolysis experiments). l-amino acids were dissolved in Milli-Q water away from light before use. Unless otherwise stated, to collect more signals, amino acids were added to the *cis* chamber to a high final concentration of 100 μM (except 5 μM, 200 μM, and 2 μM for H, P and C, respectively).

### Detection of amino acids from peptide hydrolysate

For real-time monitoring of peptide hydrolysis, peptide EAFNL or LNFAE was dissolved in Milli-Q water (2 mM) and was added to the *cis* chamber, to a final concentration of 20 μM. After recording the current trace for more than 10 min, 10 μl 16.7 U carboxypeptidase A1 was added to the *cis* chamber to initiate peptide hydrolysis. The Aβ peptides (17–27 aa) were hydrolyzed using bacterial leucyl aminopeptidase. The mixture containing 1.8 mM peptide and 5 U ml^–1^ aminopeptidase was incubated at 37 °C for 15.5 h and then heat-inactivated at 90 °C for 5 min. The hydrolysate was ultrafiltered through a filter with a 10 kDa molecular weight cut-off. Thirty microliters of filtrate was added to the *cis* chamber for detection. For each of the neoantigen peptides, angiotensin I, α-bag cell peptide (1–9 aa) and ACTH (18–39 aa), the peptide was dissolved in Milli-Q water to a final concentration of 2 mM. Eight microliters of peptide solution was mixed with 2 μL 3.3 U carboxypeptidase A1 and incubated at 37 °C for 15 min, and then the product was added to the *cis* chamber without ultrafiltration. All peptides were hydrolyzed and detected separately in independent experiments.

### Electrophysiology recording

Single-channel current recordings were amplified using an Axopatch 200B amplifier (Molecular Devices) and filtered with a built-in four-pore low-pass Bessel filter at 2 kHz. Data were digitized by a Digidata 1550B converter (Molecular Devices) at a sampling rate of 100 kHz. The data were collected by Clampex 10.2 and processed in OriginPro (2021) and R (4.0.1) software. Unless otherwise stated, all electrophysiology recordings were performed using a buffer composed of 1 M KCl and 10 mM MOPS, pH 7.5, and applied voltage of +50 mV at room temperature (23 ± 2 °C).

### Signal extraction for amino acid translocation event

To reduce the noise of raw current recording, we calculated the optimal change points of raw current, according to the mean and variance and polished the current recording using the average current of each segment time range according to the identified change points. Then we extracted the translocation events from the polished signal on the basis of the minimum blockade threshold value (0.1) against the baseline current. For all the extracted events, we calculated the blockade, dwell time and s.d. of signal current. In addition, to better describe the characteristics of each signal, we uniformly extracted the density values of 1,000 points from the density curve of the standardized current (signal current divided by *I*_0_) of each signal as the feature values of the signal (Fig. 3a). The 1,000 feature values and other calculated features, such as blockade and dwell time, were used for subsequent calculation of the signal distance and training of the machine-learning model.

### Raw signal filtering based on similarity with background noise

For the original signals of each independent experiment, we randomly selected the same number of noise signals from the corresponding blank control experiment to calculate the Euclidean distance matrix using the extracted feature values. Then we used the KNN algorithm to filter out the original signals that have any background signal among the ten nearest signals.

### Classification model training

We developed a machine-learning algorithm to automatically predict the corresponding amino acid from the signal of a translocation event. The strategy was to have the algorithm to ‘learn’ from the labeled training data set and to build an optimum classification model to recognize unlabeled events. To train the model, the blockade, dwell time, s.d. value and estimated feature value from the density curve of the standardized signal were calculated using R, to form a feature matrix (Fig. 3a). For each amino acid, we randomly selected one of the independent experiments as the validation data set, and then randomly selected 80% of all the remaining signals for the training data set. For Gly, Ala, Lys, Cys, His and Pro, the original signals were less than 1,000, so we increased the training data to 1,000 through upsampling; for amino acids with more than 1,000 original signals, we randomly selected 1,000 signals as training data without any replacements. Finally, all signals that were not used as training data or for validation were used as test set data (Supplementary Fig. 10). Model training was performed using the R package caret. A set of classifiers including RF, NB, KNN, bagged CART, AdaBoost and NNet was tested. To prevent the overfitting of model training, tenfold cross-validation was performed for each model to determine the cross-validation accuracies.

### Signal analysis for peptide hydrolysate

The feature values were extracted from the raw signals and used to predict their amino acid type with a trained RF classification model. We retained only signals with predicted probabilities higher than 0.95, to get more robust prediction results. In the real-time hydrolysis experiment, we normalized the identified number of each type of amino acid by the mean signal frequency (Fig. 2c) to get a correct abundance of each type of amino acid. To assess the similarity of different peptides, we extracted the density values of normalized current amplitude from all signals of each peptide as its feature values (Supplementary Fig. 17). Because peptides with different products have specific density curves, these extracted density values can be used to distinguish different peptides (Supplementary Fig. 17). Therefore, we calculated the Euclidean distance of all peptides from the high-dimensional feature matrix to assess the similarity between all peptides (Supplementary Fig. 18). Then, the classical MDS algorithm was used to get the best-fitting representation from the *k*-dimensional (where the *k* is the number of peptides) Euclidean distances matrix.

### Reporting summary

Further information on research design is available in the Nature Portfolio Reporting Summary linked to this article.

## Online content

Any methods, additional references, Nature Portfolio reporting summaries, source data, extended data, supplementary information, acknowledgements, peer review information; details of author contributions and competing interests; and statements of data and code availability are available at 10.1038/s41592-024-02208-7.

### Supplementary information

Supplementary Information Supplementary Materials, Tables 1–3, Figs. 1–18 and Discussion 1.

Reporting Summary

### Source data

Source Data Fig. 1 Statistical source data.

Source Data Fig. 2 Statistical source data.

Source Data Fig. 3 Statistical source data.

Source Data Fig. 4 Statistical source data.

Source Data Fig. 5 Statistical source data.

Source Data Fig. 6 Statistical source data.

## Data Availability

The data sets generated and/or analyzed in this study are available within the source data. All the data supporting the findings of this study are available at 10.6084/m9.figshare.24968331. Source data are provided with this paper.

## Code availability

We developed an R package named AANanopore to process and extract the amino acid signals from the raw ABF file. The experimental data were analyzed using R (version 4.0.1). The AAnanopore package, codes, algorithms used in this study and the demo data and user manual are available at https://zenodo.org/doi/10.5281/zenodo.10644198.
