# Supplementary material for: Real-time detection of 20 amino acids and discrimination of pathologically relevant peptides with functionalized nanopore
Source: Nat Methods. 2024 Mar 5;21(4):609–18. doi: 10.1038/s41592-024-02208-7 (PMC11009107; doi:10.1038/s41592-024-02208-7)
Supplement: Supplementary file 1 — Supplementary Materials, Tables 1–3, Figs. 1–18 and Discussion 1. [file 41592_2024_2208_MOESM1_ESM.pdf]

nature methods

Article https://doi.org/10.1038/s41592-024-02208-7

Real-time detection of 20 amino acids and discrimination of pathologically relevant peptides with functionalized nanopore

In the format provided by the authors and unedited

Materials

The plasmid for M2MspA-N91H was prepared by Genewiz (China). *E. coli* BL21 (DE3) competent cell, lysogeny broth (LB), kanamycin and isopropyl β-D-1-thiogalactopyranoside (IPTG) were purchased from Sangon Biotech (China). 1,2-diphytanoyl-sn-glycero-3-phosphocholine (DPhPC) was purchased from Avanti Polar Lipids (USA). Potassium chloride (KCl), 3-morpholino propionic acid (MOPS) and copper chloride were from BBI life sciences corporation (China). All twenty proteinogenic amino acids were purchased from Aladdin (China). O-phosphoryl-L-serine (P-S) was provided by Sigma-Aldrich (USA), Nε-acetyl-L-lysine (Ac-K) and S-carboxymethyl-L-cysteine (CMC) were purchased from Tokyo Chemical Industry (Japan). All peptides were synthesized by Sangon Biotech (China). Carboxypeptidase A1 and bacterial leucyl aminopeptidase were purchased from Sigma-Aldrich (USA).

Supplementary Tables

Supplementary Table 1. The duration of baseline current (I_0) during measurement[a].

Independent experiments	t_{record} (min)	t_0 (min)	t_0/t_{record}
#1	5.04	4.52	0.897
#2	5.00	4.29	0.858
#3	5.00	4.53	0.906
#4	5.00	4.50	0.900
#5	5.00	4.18	0.836

[a] Total time of a continuous recording (t_{record}). The added-up time in t_{record} when the current stabilized around I_0.

Supplementary Table 2. Statistics of blockade, dwell time and signal frequency of events of twenty proteinogenic amino acids.

Amino acids	Blockade (mean ± s.d.)	Dwell time (ms) (mean ± s.e.)	Signal frequency (μM^{-1} min^{-1}) (mean ± s.d.)
Ala	0.147 ± 0.0031	2.39±0.15	1.503 ± 1.043
Arg	0.170 ± 0.0029	2.10±0.03	2.052 ± 1.112
Asn	0.165 ± 0.0034	2.69±0.33	19.721 ± 14.004
Asp	0.215 ± 0.0038	4.13±0.20	6.361 ± 3.854
Cys	0.209 ± 0.0062	3.07±0.36	64.634 ± 53.678
Gln	0.189 ± 0.0029	2.87±0.13	8.892 ± 7.223
Glu	0.244 ± 0.0041	3.23±0.19	12.524 ± 7.658
Gly	0.119 ± 0.0022	2.01±0.11	1.303 ± 1.480
His1	0.248 ± 0.0017	42.7±17.15	10.615 ± 1.982
His2	0.237 ± 0.0066	1.96±0.32	10.615 ± 1.982
Ile	0.208 ± 0.0032	2.22±0.06	3.164 ± 1.576
Leu	0.200 ± 0.0026	1.93±0.09	3.373 ± 2.107
Lys	0.171 ± 0.0026	2.79±0.35	0.862 ± 0.530
Met	0.198 ± 0.0031	2.32±0.14	8.634 ± 5.144
Phe	0.220 ± 0.0044	2.27±0.07	11.036 ± 3.485
Pro	0.219 ± 0.0028	7.95±0.44	0.195 ± 0.111
Ser	0.132 ± 0.0033	1.64±0.08	12.404 ± 6.133
Thr	0.161 ± 0.0031	2.74±0.14	13.484 ± 10.064
Trp	0.227 ± 0.0029	8.83±0.54	5.954 ± 3.496
Tyr	0.213 ± 0.0057	3.97±0.48	4.893 ± 3.701
Val	0.192 ± 0.0036	2.51±0.16	2.240 ± 1.162

Supplementary Table 3. Statistics of discrimination of twenty proteinogenic amino acids using machine learning algorithm.

	Sensitivity	Specificity	Precision	Recall	F1	Balanced Accuracy
Ala	1.0000	0.9995	0.9706	1.0000	0.9851	0.9998
Arg	0.5563	0.9926	0.7304	0.5563	0.6316	0.7744
Asn	0.6582	0.9866	0.7702	0.6582	0.7098	0.8224
Asp	0.7403	0.9828	0.4385	0.7403	0.5507	0.8615
Cys	0.5000	0.9977	0.3333	0.5000	0.4000	0.7488
Gln	0.7740	0.9954	0.8782	0.7740	0.8228	0.8847
Glu	0.9912	0.9998	0.9912	0.9912	0.9912	0.9955
Gly	0.9545	1.0000	1.0000	0.9545	0.9767	0.9773
His	0.9792	0.9998	0.9792	0.9792	0.9792	0.9895
Ile	0.9080	0.9922	0.7054	0.9080	0.7940	0.9501
Leu	0.7023	0.9388	0.5000	0.7023	0.5841	0.8206
Lys	0.5000	0.9918	0.3750	0.5000	0.4286	0.7459
Met	0.5041	0.9722	0.7512	0.5041	0.6033	0.7381
Phe	0.8438	0.9859	0.9284	0.8438	0.8840	0.9148
Pro	0.7059	0.9951	0.3636	0.7059	0.4800	0.8505
Ser	1.0000	0.9997	0.9987	1.0000	0.9994	0.9999
Thr	0.8493	0.9803	0.6019	0.8493	0.7045	0.9148
Trp	0.8848	0.9928	0.8914	0.8848	0.8881	0.9388
Tyr	0.8901	0.9974	0.8804	0.8901	0.8852	0.9438
Val	0.8194	0.9768	0.6507	0.8194	0.7254	0.8981

Supplementary Figures

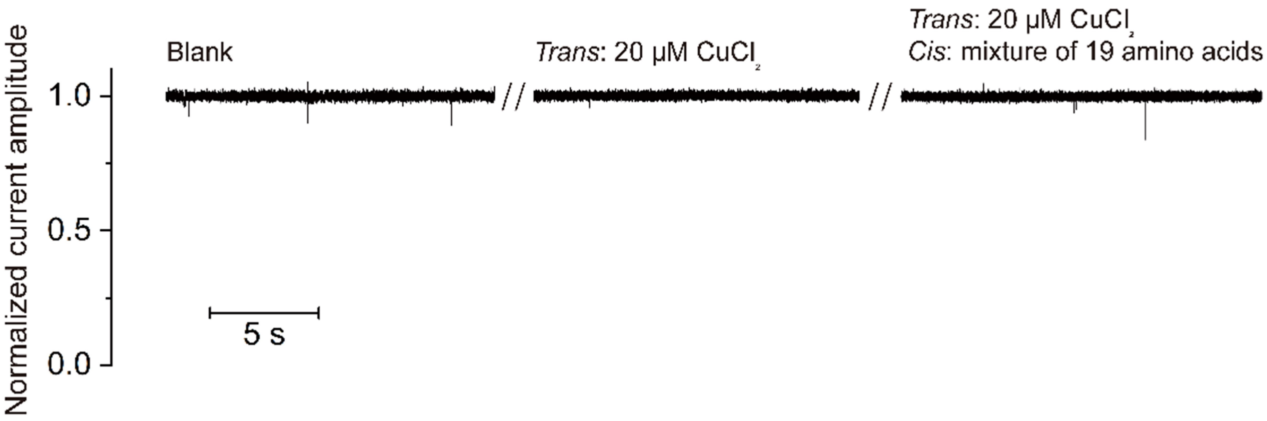

Supplementary Figure 1. Detection of the mixture of 19 amino acids using M2MspA. Copper chloride and amino acids were added to the same nanopore successively. No binding event of Cu^{2+} and amino acids were observed. The experiments were conducted in 1 M KCl, 10 mM MOPS, pH 7.5. The voltage applied was +50 mV.

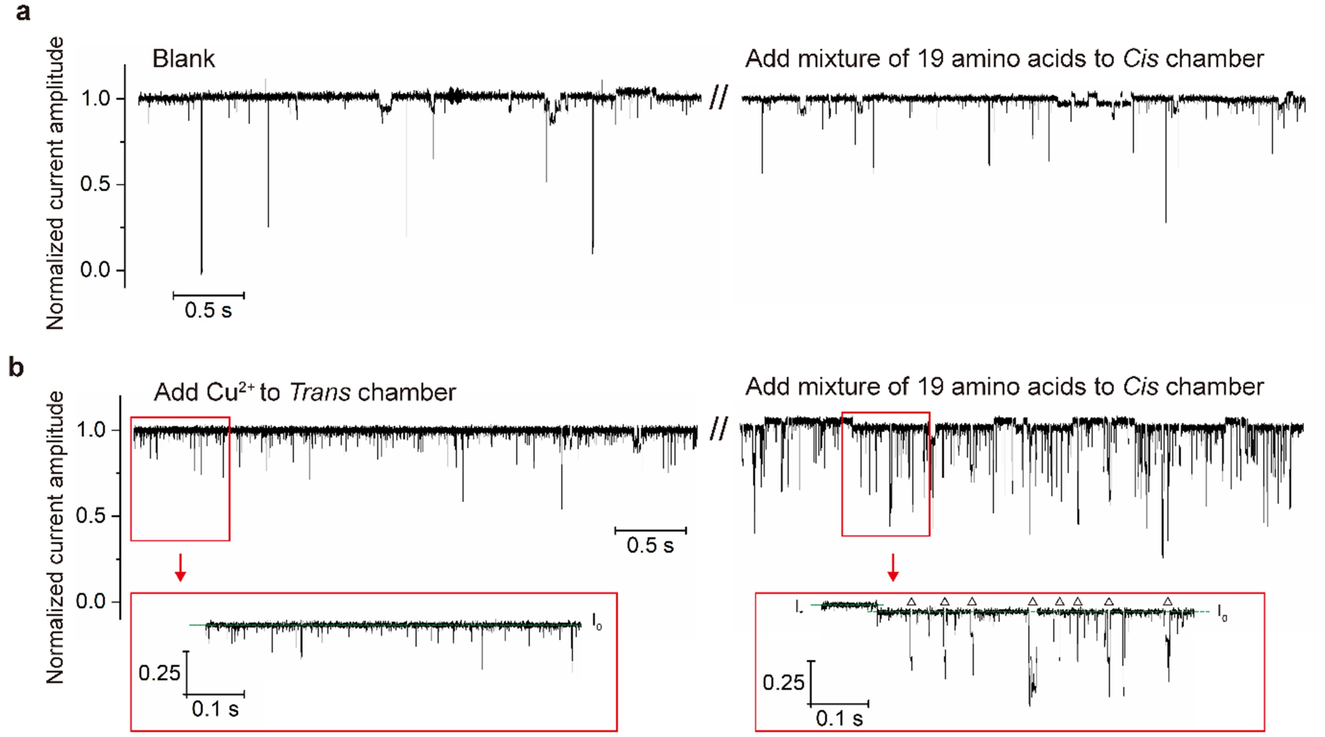

Supplementary Figure 2. Detection of the mixture of 19 amino acids using M2MspA-N91H without Cu^{2+}. a, no binding event of amino acids was observed if Cu^{2+} was not added. **b**, amino acids were detected when Cu^{2+} was added. The triangles represent the signals of amino acids. The experiments were conducted in 1 M KCl, 10 mM MOPS, pH 7.5. The voltage applied was +50 mV.

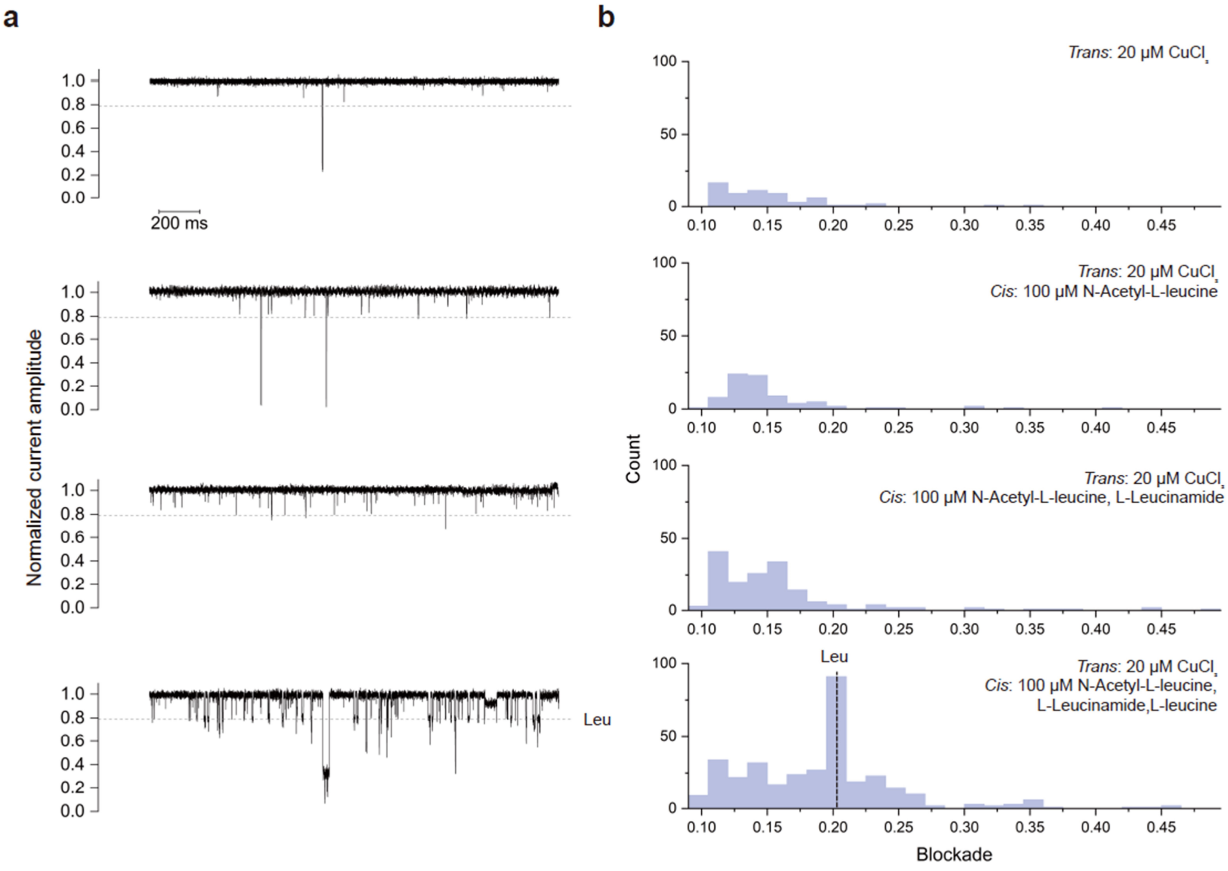

Supplementary Figure 3. Detection of acetylated leucine, amidated leucine, and leucine. a, representative current traces from a single pore after successively adding Cu^{2+}, N-Acetyl-L-leucine, L-leucinamide, and L-leucine (from top to bottom); **b**, histograms of the blockade of translocation events. The events of each histogram were extracted from 30 seconds of the current trace. Only after the addition of L-leucine, the corresponding translocation events were observed. The results indicated that L-leucine could not coordinate Cu^{2+} without α-carboxyl group or α-amine group. The experiments were conducted in 1 M KCl, 10 mM MOPS, pH 7.5. The voltage applied was +50 mV.

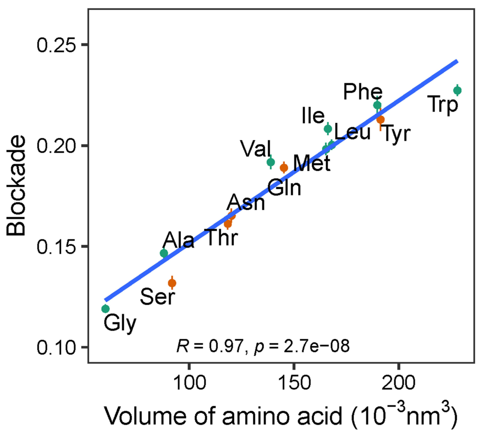

Supplementary Figure 4. Scatter plot of volume versus blockade of amino acids. Compared with Figure 2b, proline, cysteine, and amino acids with charged side chain are excluded. The Pearson correlation coefficient between the mean blockade and volume reaches up to 0.97. For each amino acid, the mean blockade and its standard deviation were calculated from the Gaussian fitting result of data points ((n = 7166 (F), 3934 (W), 2768 (Y), 3025 (I), 8004 (M), 8131 (T), 8101 (S), 3750 (L), 857 (A), 1149 (G), 7873 (Q), 9634 (N), 2119 (V)) from at least three independent experiments. The R and p in this plot represent the Pearson correlation coefficient and p-value respectively.

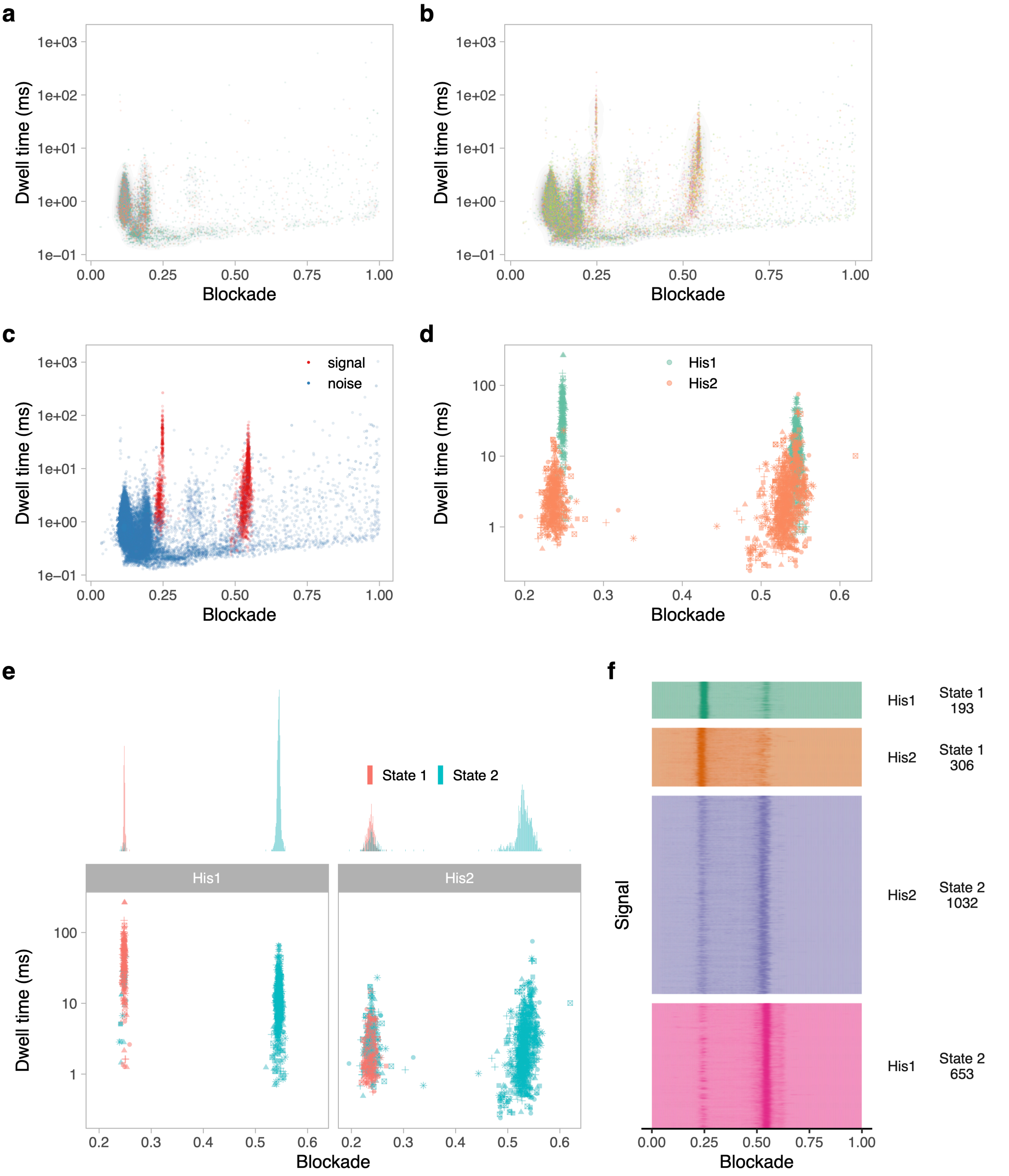

Supplementary Figure 5. Signal extraction and sub-type classification of histidine (His). a, scatter plot of background signals from blank control without any amino acids. The colour of the dots represents different replicate experiments, and the shade of the colour represents the density of dots at that location. **b**, scatter plot of the identified signals after the addition of histidine. **c**, we used the K-Nearest Neighbour (KNN) algorithm to filter out the original signals that have any background signal among the 10 nearest signals (described in Method section). The red dots in the scatter plot are the histidine signals (n = 2,184),

and the blue dots are the noise (n = 13,514). **d**, based on the noticeable difference in dwell time and blockade, we found that histidine has two different states. We used the K-means clustering method to successfully distinguish histidine signals into two sub-types: His1 (n = 846) and His2 (n = 1,338). The His1 has a longer dwell time and a higher blockade than His2. The different shapes of the dots in the scatter plot represent different repeated experiments. **e**, according to the normalized current density of the signal, both His1 and His2 can be divided into two types: state 1 signals and state 2 signals. Figure e is a scatter plot of the blockade and dwell time of two different signals, state 1 and state 2, of His1 and His2. **f**, Density plots of normalized currents in different states, state 1 and state 2, of His1 and His2.

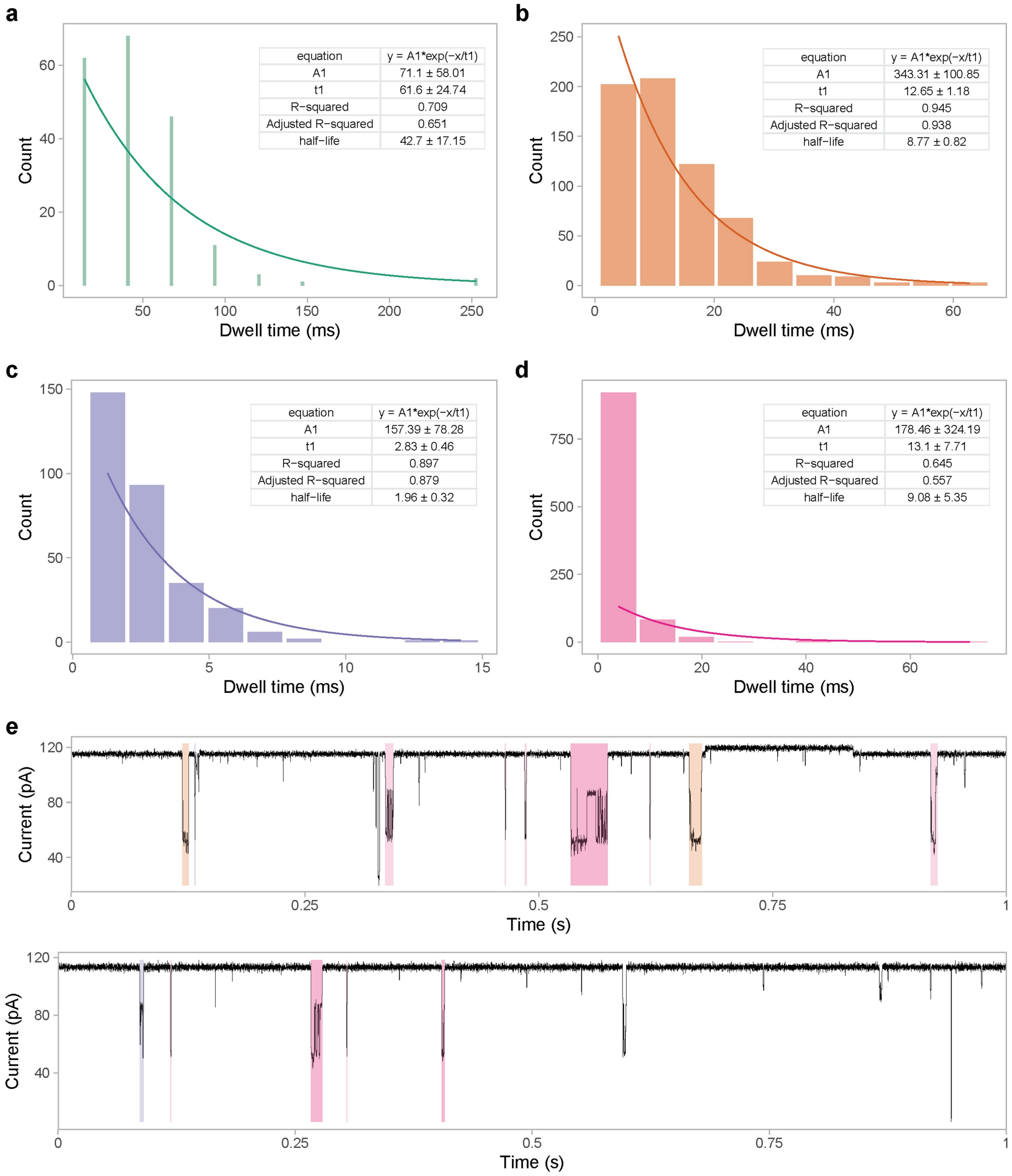

Supplementary Figure 6. Fitting of the exponential decay function of the dwell time of the histidine signal. a-d, the histogram of dwell time and fitted exponential decay function of state 1 of His1, state 2 of His1, state 1 of His2, and state 2 of His2 respectively. The fitted parameters of the exponential decay function, half-life, R-squared, and adjusted R-squared are shown in tabular form at the top of each graph (mean ± se (standard error)). **e**, a representative trace containing different types (His1 and His2) and different states (state 1 and state 2) of histidine sensing events. The green, orange, purple, and red rectangles, corresponding to Figure **a-d**, indicated the signal of state 1 of His1, state 2 of His1, state 1 of His2, and state 2 of His2 respectively.

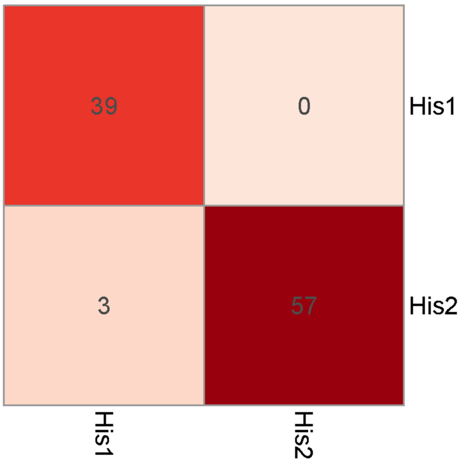

Supplementary Figure 7. The confusion matrix of random forest model of different histidine types in testing data set. We used the extracted feature matrix (See Method) of His1 and His2 to train a random forest model for histidine sub-type classifying. The model achieved an accuracy of 97% on the test set.

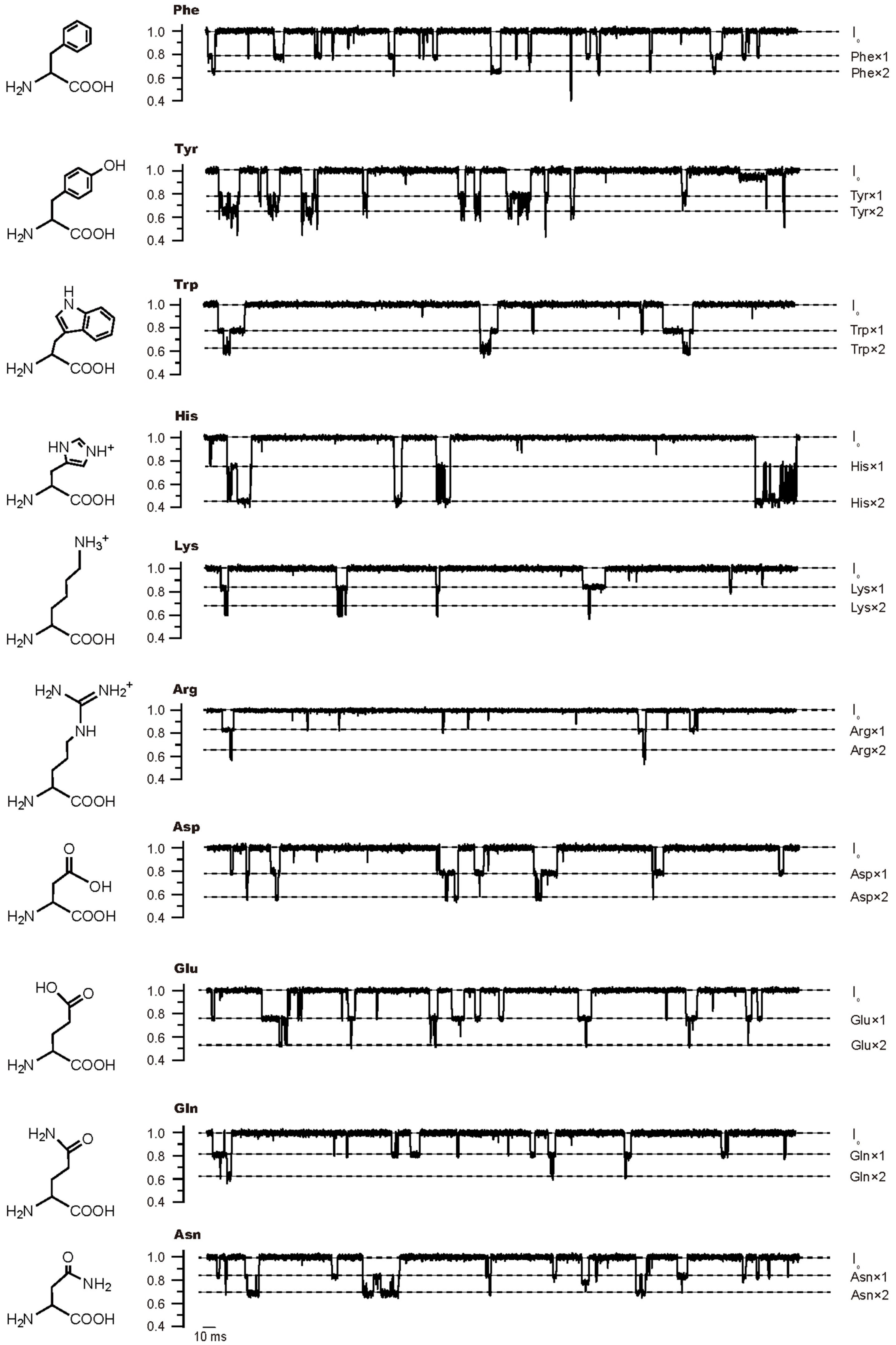

Phe
1.0
0.8
0.6
0.4
I0
Phe×1
Phe×2
H2N
COOH
Tyr
OH
Tyr×1
Tyr×2
Trp
Trp×1
Trp×2
His
HN
NH+
His×1
His×2
Lys
NH3+
Lys×1
Lys×2
Arg
H2N
NH2+
NH
Arg×1
Arg×2
Asp
Asp×1
Asp×2
Glu
HO
Glu×1
Glu×2
Gln
Gln×1
Gln×2
Asn
NH2
Asn×1
Asn×2
10 ms

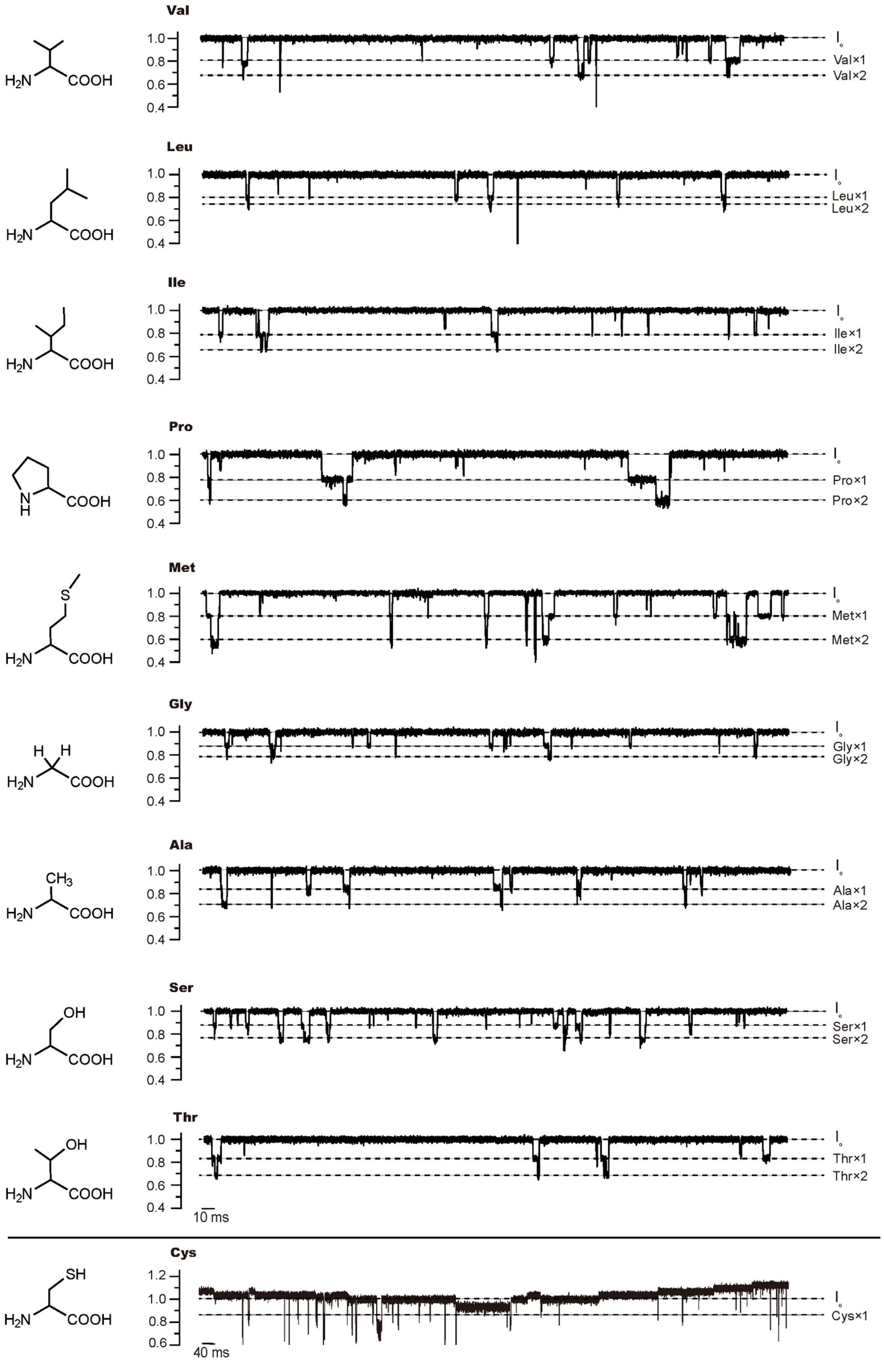
Val
Leu
Ile
Pro
Met
Gly
Ala
Ser
Thr
Cys
H2N COOH
1.0
0.8
0.6
0.4
1.2
I0
Val×1
Val×2
Leu×1
Leu×2
Ile×1
Ile×2
Pro×1
Pro×2
Met×1
Met×2
Gly×1
Gly×2
Ala×1
Ala×2
Ser×1
Ser×2
Thr×1
Thr×2
Cys×1
10 ms
40 ms

Supplementary Figure 8. Representative current signatures of twenty proteinogenic amino acids. The y-axis represents the normalized current amplitude. Current baseline (I_0), the corresponding current states for the binding of one amino acid (AA×1, state 1) and two same amino acids (AA×2, state 2) are indicated by a dashed line. The signals generated from multiple binding events (AA×2) show different patterns among amino acids, which could be related to the R group. The fluctuation of open pore current after the addition of cysteine suggests a strong interaction between the sulfhydryl group of cysteine and the copper-nanopore complex (The bottom panel). The final concentration of each amino acid added here is 100 μM (except 5 μM, 190 μM and 2 μM for H, P, and C, respectively)

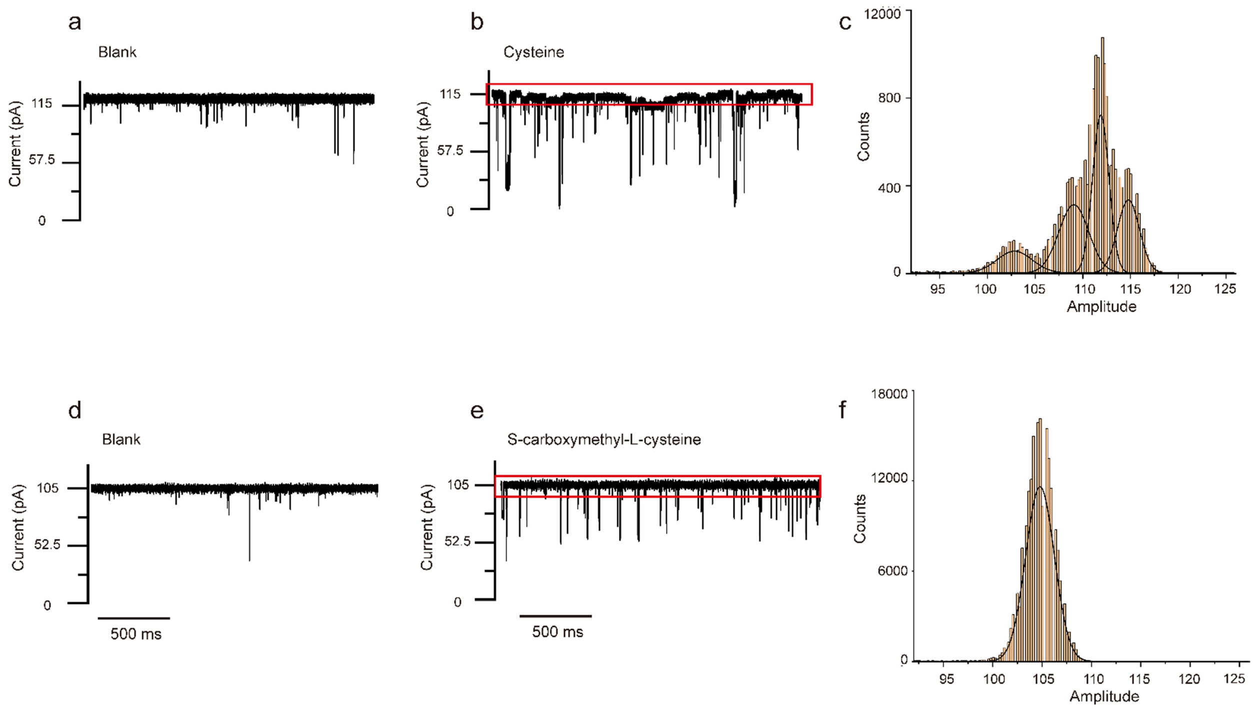

Supplementary Figure 9. Current baselines change during detection of cysteine and S-carboxymethyl-L-cysteine. a-b, representative current traces before and after the addition of cysteine. **c**, histogram of current amplitude for cysteine detection. **d-e**, representative current traces before and after the addition of S-carboxymethyl-L-cysteine. **f**, histogram of current amplitude for S-carboxymethyl-L-cysteine detection. The current traces exhibit pronounced instability following the addition of cysteine, with no such phenomenon observed upon the addition of S-carboxymethyl-L-cysteine. This difference is evident in the histogram, where multiple peaks are discernible in the current baseline following the introduction of cysteine, reflecting substantial fluctuations in current. In contrast, the addition of S-carboxymethyl-L-cysteine results in a single peak, indicating the absence any adverse impact on current baseline stability.

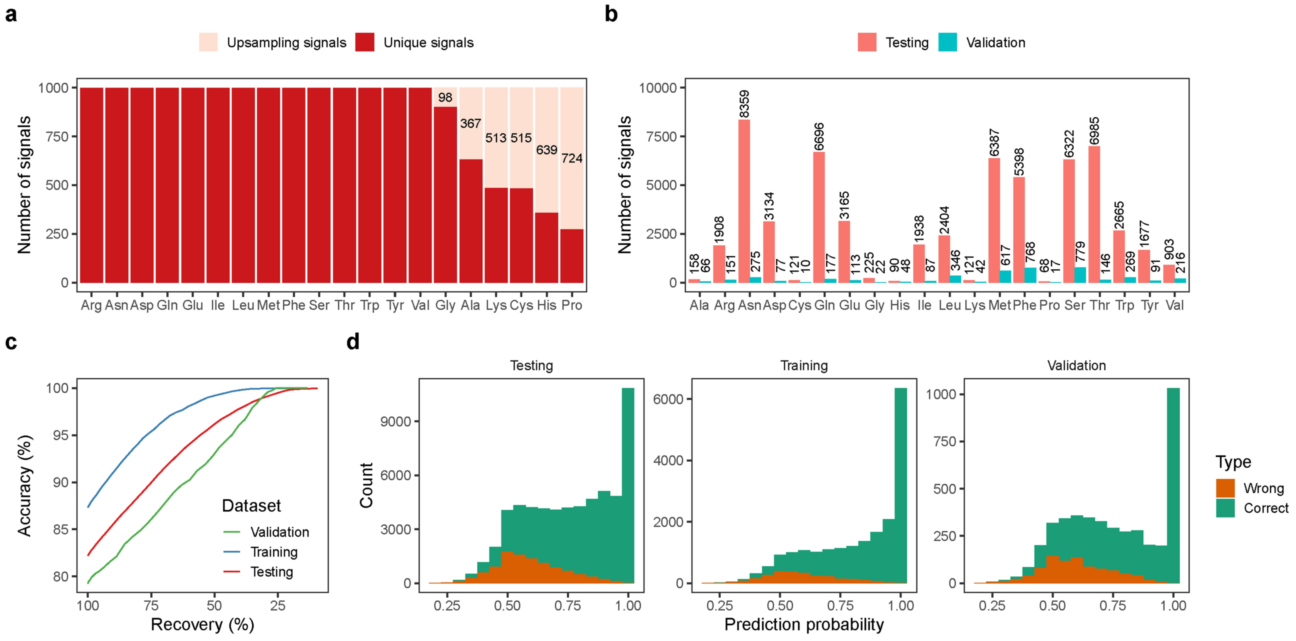

Supplementary Figure 10. The input data and performance of random forest models. a, the number of input signals of each amino acid for model training. In order to balance the number of different types of amino acids in the training set, for the amino acids Gly, Ala, Lys, Cys, His, and Pro, since the original signal is less than 1000, we increased the training data to 1000 through up-sampling. **b**, the number of signals of each amino acid used by testing and validation data set. **c**, The trade-off between classification accuracy and signal recovery of RF classifier in training, testing, and validation dataset. **d**, The RF classifier prediction probability distribution of correctly and incorrectly labeled signals within the training, testing, and validation dataset.

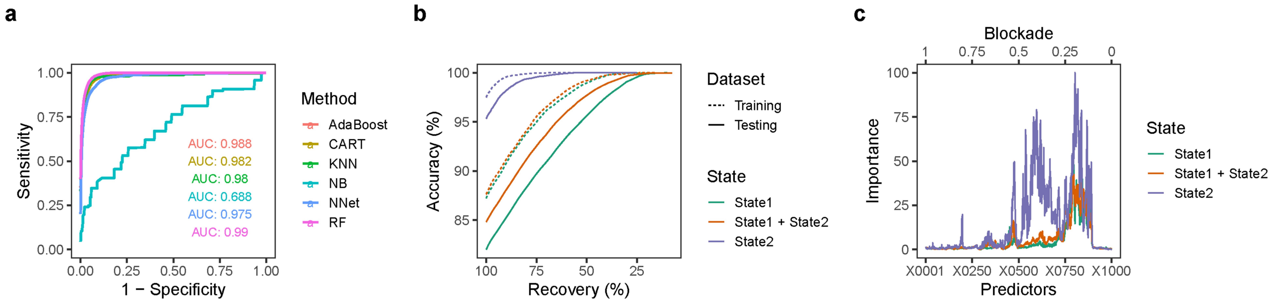

Supplementary Figure 11. Comparison of different machine learning algorithms and the advantage of state 2 signals in amino acids distinguishing. a, the receiver operator characteristic (ROC) curves and the area under the curves (AUC) of six different machine learning algorithms in a tiny dataset (100 signals for each amino acid). **b**, the trade-off between classification accuracy and signal recovery in training and testing dataset of state 1, state 2, or state 1 + state 2 random forest (RF) classifiers. In the state 1 or state 2 model, we only selected the state 1 or state 2 signals for model training and assessment. However, in state 1 + state 2 model, we used both of state 1 and state 2 signals. **c**, the importance of predictors of state 1, state 2, or state 1 + state 2 RF classifiers. The upper y-axis represents the corresponding blockade of each feature.

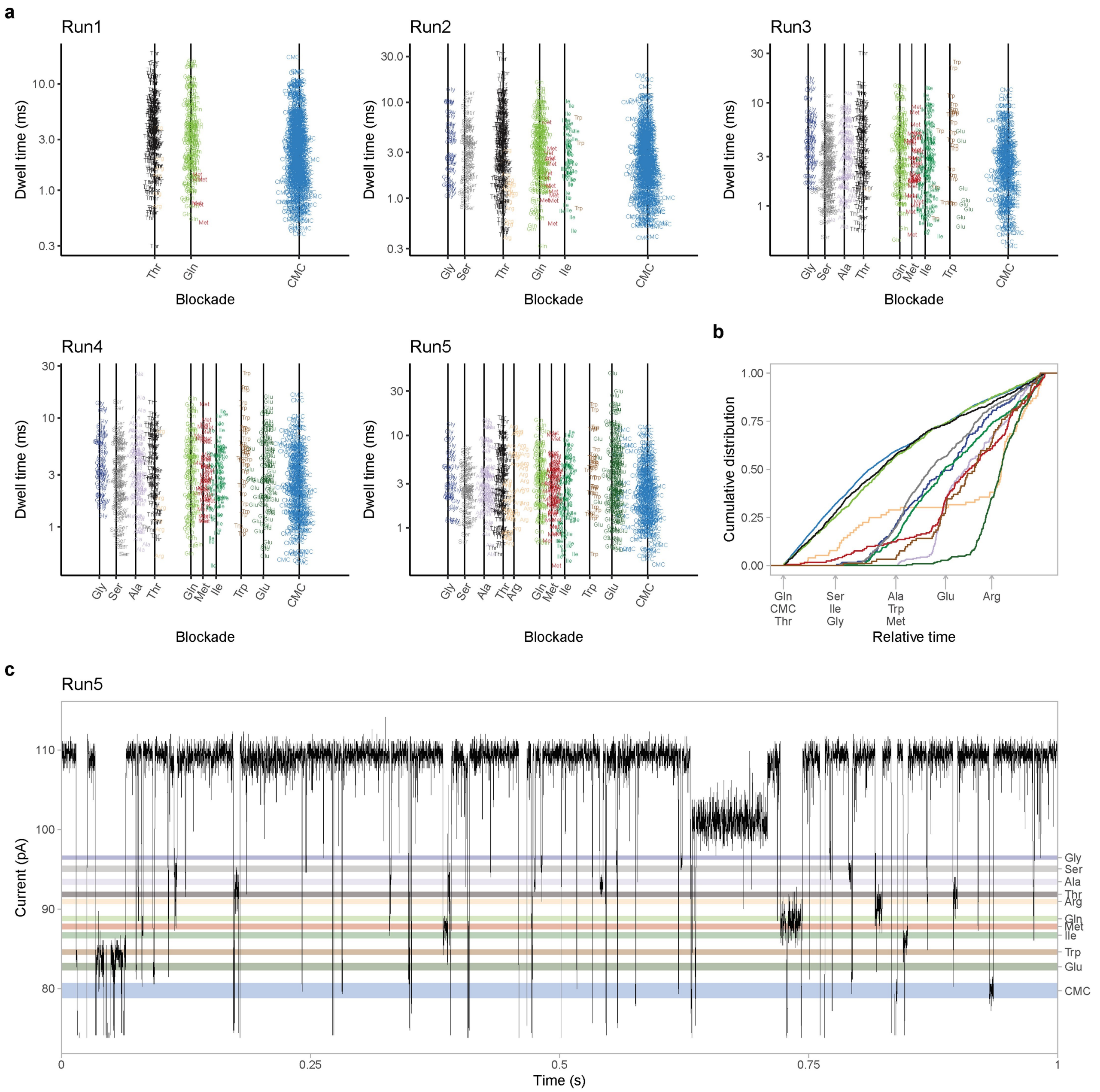

Supplementary Figure 12. Identification of the mixture containing 10 proteinogenic amino acids and S-carboxymethyl-L-cysteine (CMC). a, different types of amino acids were added successively. Thr, Gln, and CMC were added in Run1. In the second run, we newly added Gly, Ser, and Ile. In the third run, we newly added Ala, Met, and Try. In the fourth and fifth runs, we newly added Glu and Arg respectively. We extracted the raw signals and filtered the noises according to their similarity with background signals (described in the method section). Then, we predicted each signal using the trained random forest model. The scatter plot showed the prediction results of each run after noise filtering. The x-axis label and black vertical line indicated the theoretical blockade of added target amino acids. **b**, the cumulative distribution of identified amino acids according to the relative monitoring time. **c**, the current trace of real-time detection from Run5 with 11 different amino acids.

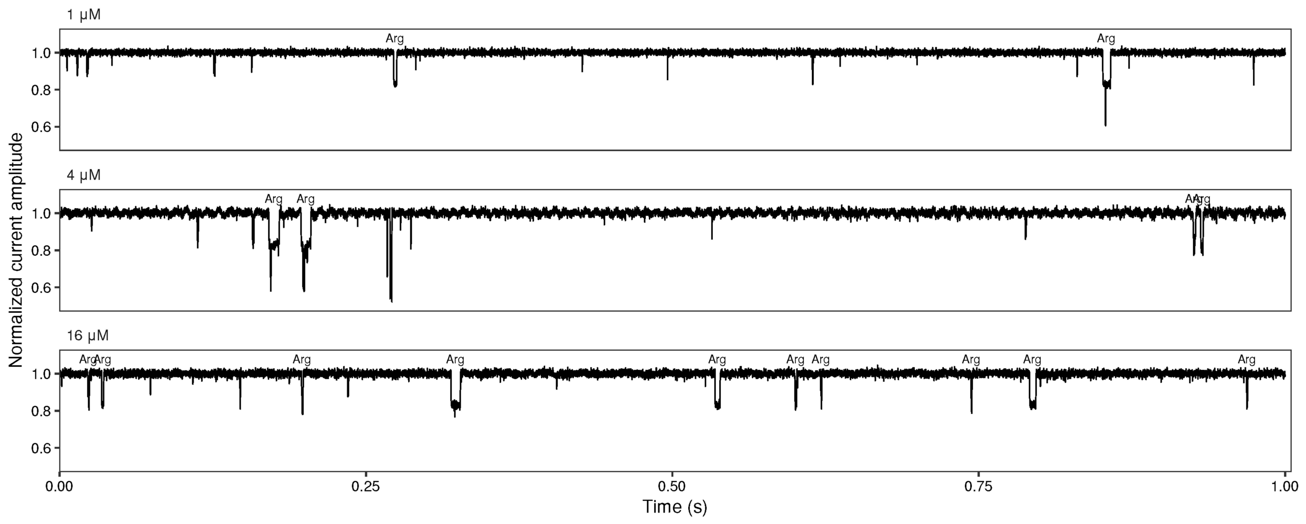

Supplementary Figure 13. Representative current traces of L-Arg detection at different concentrations. The L-Arg was added to the same nanopore to a final concentration of 1 μM, 4 μM and 16 μM (From top to bottom panel). With the increase of concentration, more signals of L-Arg were identified. The buffer used here was 1 M KCl, 10 mM MOPS, pH 7.5. The applied voltage was +50 mV.

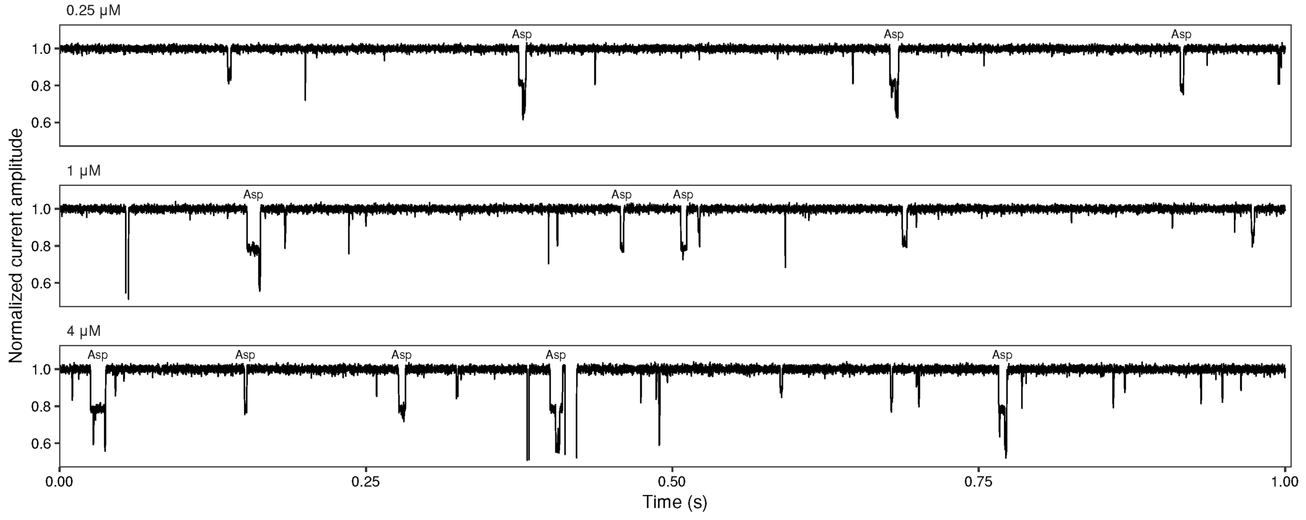

Supplementary Figure 14. Representative current traces of L-Asp detection at different concentrations. The L-Asp was added to the same nanopore to a final concentration of 0.25 μM, 1 μM and 4 μM (From top to bottom panel). The buffer used here was 1 M KCl, 10 mM MOPS, pH 7.5. The applied voltage was +50 mV.

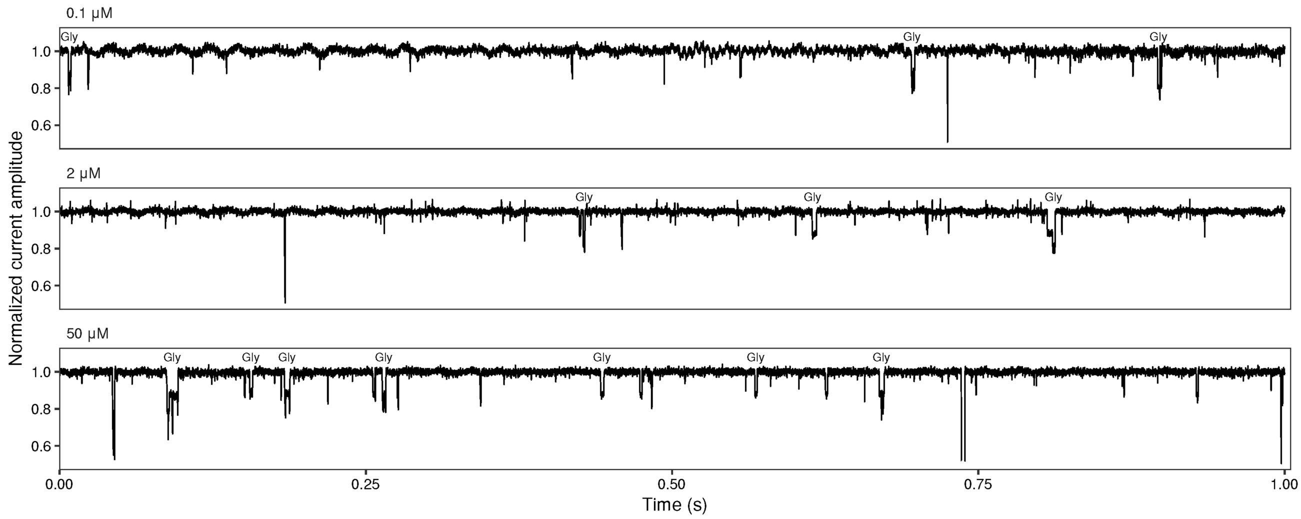

Supplementary Figure 15. Representative current traces of L-Gly detection at different concentrations. The L-Gly was added to the same nanopore to a final concentration of 0.1 μM, 2 μM and 50 μM (From top to bottom panel). The buffer used here was 1 M KCl, 10 mM MOPS, pH 7.5. The applied voltage was +50 mV.

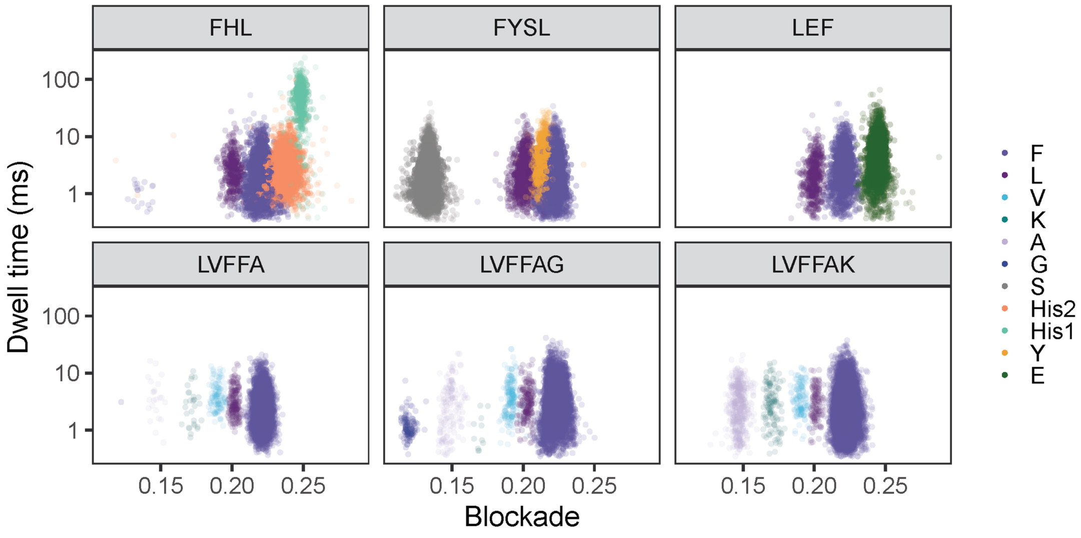

Supplementary Figure 16. Scatter plots of dwell time *versus* blockade of identified amino acids in the peptide hydrolysates by RF model. Upper panel: from left to right, the hydrolysates of Angiotensin I, α-Bag Cell Peptide (1-9) and Adrenocorticotropic hormone (18-39). Lower panel: the hydrolysates of Alzheimer's peptides

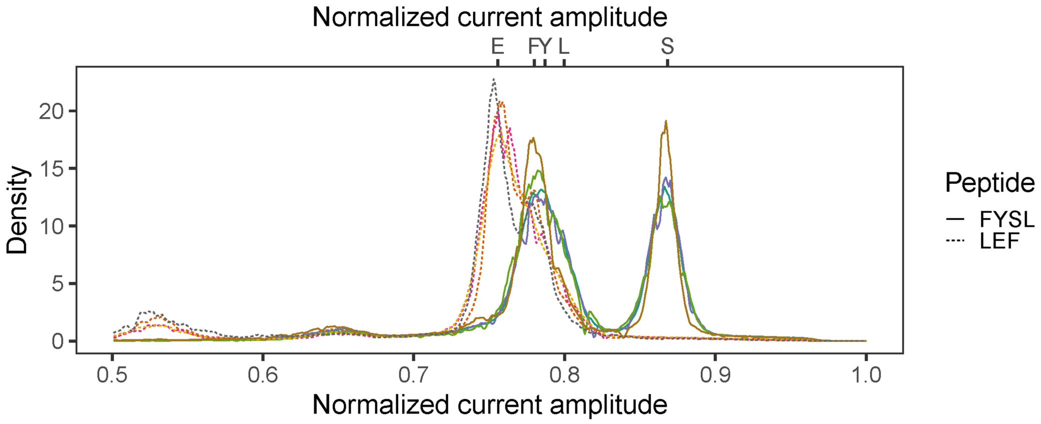

Supplementary Figure 17. The estimated density line of standardized current of all identified signals of peptide hydrolysates. The red and blue lines represent density plots of standardized currents for all signals identified from the hydrolysates of a LEF and FYSL, respectively. The gray vertical lines indicate the theoretical position of E, F, Y, L and S amino acids after standardization of blocking currents. This density curve shows the signal distribution of different amino acids in the hydrolyzed products and will be used to characterize the amino acid composition of the pre-hydrolysis polypeptide.

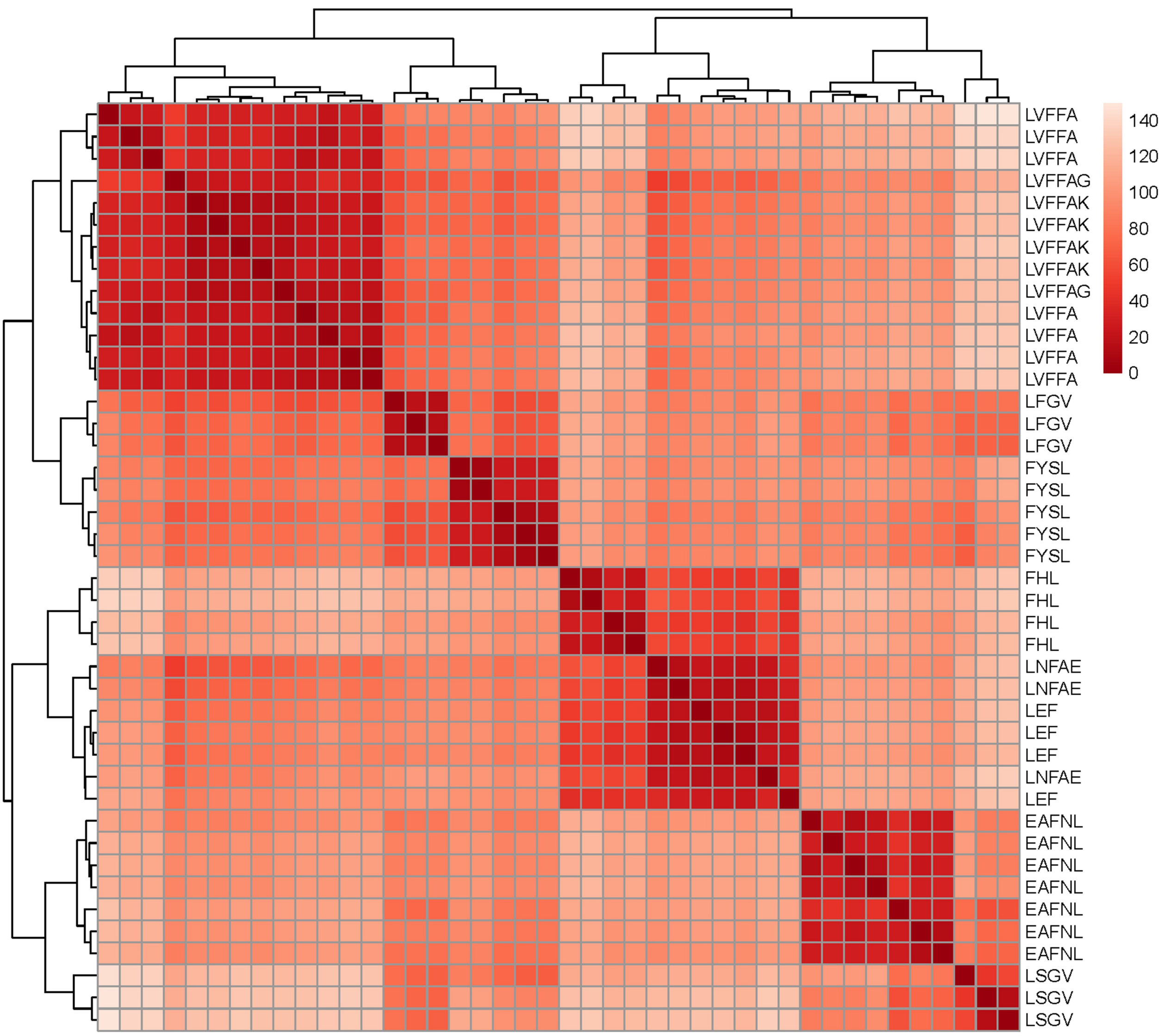

Supplementary Figure 18. The heatmap of Euclidean distance between different peptides. The Euclidean distance was calculated from the estimated density value of standardized current of all peptides.

Supplementary Discussion

Supplementary Discussion 1

“The state 1 and state 2 signal represents the binding of one amino acid and two amino acids, respectively. In the training model where only state 2 signals were used as the input data, the classification accuracy was higher than other models (Supplementary Figure. 11b). The state 2 signals were shown more robust than state 1 signals. Therefore, we included all the state 1 and state 2 signals as the input data for training (Figure 3, Supplementary Figure. 11b, c). Indeed, the addition of state 2 signals in “state 1+state 2” model improved the classification accuracy, compared with the “state 1” model. However, we did not include the state 2 signals for signal identification. It is because during the detection of amino acids mixture, the state 2 signals may result from the binding of two different amino acids, which has hundreds of combinations. These kinds of signals have not yet been completely determined by experiment.
We also observed other interesting results (Supplementary Figure 8), of which the molecular mechanism behind remains unclear. 1) The current blockade resulted from the binding of the second amino acids differed from the blockade from the first amino acid. For example, for Lys and Arg, the second blockade was larger than the first one. For leu, it was opposite. We reasoned that there could be a preference of binding site for different amino acids. Since there are four binding sites within nanopore, the second amino acid molecule can bind to the ortho- or para- positions, which may result in a blockade differing from the first blockade; 2) The concentration dependence of the frequency of state 2 signals should be analysed for all the amino acids. It could provide the evidence for the assumption that state 2 signals were generated by the simultaneous binding of two amino acids. It could also reveal the proportions of state 1 and state 2 signals at different concentrations, which helps understand the binding mechanism of two amino acids at molecular level.
